# Characterization of the small RNA component of the transcriptome from grain and sweet sorghum stems

**DOI:** 10.1186/1471-2164-12-356

**Published:** 2011-07-08

**Authors:** Martín Calviño, Rémy Bruggmann, Joachim Messing

**Affiliations:** 1 Waksman Institute of Microbiology, Rutgers University, 190 Frelinghuysen Road, Piscataway, New Jersey 08854-8020, USA

## Abstract

**Background:**

Sorghum belongs to the tribe of the *Andropogoneae *that includes potential biofuel crops like switchgrass, Miscanthus and successful biofuel crops like corn and sugarcane. However, from a genomics point of view sorghum has compared to these other species a simpler genome because it lacks the additional rounds of whole genome duplication events. Therefore, it has become possible to generate a high-quality genome sequence. Furthermore, cultivars exists that rival sugarcane in levels of stem sugar so that a genetic approach can be used to investigate which genes are differentially expressed to achieve high levels of stem sugar.

**Results:**

Here, we characterized the small RNA component of the transcriptome from grain and sweet sorghum stems, and from F2 plants derived from their cross that segregated for sugar content and flowering time. We found that variation in miR172 and miR395 expression correlated with flowering time whereas variation in miR169 expression correlated with sugar content in stems. Interestingly, genotypic differences in the ratio of miR395 to miR395* were identified, with miR395* species expressed as abundantly as miR395 in sweet sorghum but not in grain sorghum. Finally, we provided experimental evidence for previously annotated miRNAs detecting the expression of 25 miRNA families from the 27 known and discovered 9 new miRNAs candidates in the sorghum genome.

**Conclusions:**

Sequencing the small RNA component of sorghum stem tissue provides us with experimental evidence for previously predicted microRNAs in the sorghum genome and microRNAs with a potential role in stem sugar accumulation and flowering time.

## Background

Small RNAs (18-25 nt) regulate many developmental and physiological processes in plants through the regulation of gene expression at either the transcriptional or post-transcriptional level [1-3]. They can be subdivided into short-interfering RNAs (siRNAs) and microRNAs (miRNAs) [3-5].

MicroRNAs are derived from capped and polyadenylated primary (pri)-miRNA transcripts that are transcribed by RNA polymerase II and can form a hairpin-loop structure by intramolecular pairing [4,6]. Two sequential cleavages mediated by DICER LIKE 1 (DCL1) are required to produce a mature miRNA [4,7]. In the first cleavage, DCL1 cleaves near the base of the hairpin-loop stem of the pri-miRNA to produce a miRNA precursor (pre-miRNA). The second cleavage takes place near the loop of the pre-miRNA to produce a miRNA/miRNA* duplex. The mature miRNA is then loaded into the RNA-induced silencing complex (RISC) and can guide the sequence-specific cleavage or translational inhibition of target mRNAs [2,4,7,8], as well as gene silencing through DNA methylation [9,10], whereas the non-incorporated miRNA* strand is usually degraded.

Through the use of next-generation sequencing, the small RNA component of the *Arabidopsis *and rice transcriptomes has been well characterized, more than in any other plant species [11,12]. This is reflected in the miRBase database (http://www.mirbase.org, release 16: September 2010), where 213 miRNAs are described for *Arabidopsis *whereas 462 miRNAs are described for rice. Besides rice, the identification of miRNAs through deep sequencing in other grasses including maize, wheat, and *Brachypodium *have been described [13-15]. The identification of rice, maize and wheat miRNAs from different tissues, developmental stages and stress-treatments [12,13,15-20], provides an opportunity to understand how miRNAs regulate the expression of genes influencing traits of agronomic importance. Currently, a trait of particular relevance for biofuel production is that of sugar accumulation in the stem of sorghum [*Sorghum bicolor *(L.) Moench] and sugarcane (*Saccharum spp*.), two closely related C4 grasses that diverged from each other about 8-9 million years ago [21].

In both species, sucrose is the main type of sugar and accumulates in the parenchyma tissue of the juicy stems [22,23]. High sucrose content is a highly desirable trait since the accumulated sugar can be fermented to produce bioethanol as a source of renewable energy [24]. Although sugarcane has been extensively used as a source of biofuel, its use as a model system to understand the genetics of sugar accumulation is hampered by its complex genome, with several cultivars differing greatly in their ploidy levels [25]. Sorghum instead, is a diploid species and its genome has been recently sequenced [26]. In addition, the intra-species variation for sugar content is much more pronounced in sorghum than in sugarcane [27], with sorghum cultivars known as sweet sorghums accumulating high levels of sugars relative to grain sorghums [28]. This makes sorghum a more suitable system to study the genetic basis of sugar accumulation. Still, the gene repertoire involved in sugar accumulation is not well characterized in sorghum due to the low heritability of the trait and its quantitative inheritance. In addition, previous reports have suggested the existence of trade-offs between sugar content and other plant traits such as flowering time [28,29].

We also observed that sugar accumulation (measured as Brix degree and referred herein as Brix) in the stem of grain sorghum BTx623 and sweet sorghum Rio cultivars differed at the time of flowering. Interestingly, 80% of the differentially expressed genes in stem tissue between the two cultivars had orthologous counterparts in syntenic positions in rice [30,31]. This suggested that the ability of sorghum to accumulate soluble sugars relative to rice could not be explained by differences in their gene content but rather due to gene regulation at either the transcriptional or post-transcriptional level. To address the latter possibility, we characterized the small RNA portion of transcriptomes derived from stem tissues of grain and sweet sorghum in order to investigate the microRNA-mediated regulation of genes involved in sugar accumulation and flowering time. Using the SOLiD next generation sequencing system, we sequenced with an unprecedented depth small RNAs libraries from BTx623 and Rio, and from a pool of selected F2 plants derived from their cross that differed in sugar content and flowering time. We also reasoned that plant stems would provide us with a representative tissue to experimentally validate the previously predicted miRNAs of the sorghum genome [26]. Indeed, we not only detected the expression of 25 miRNA families from the 27 predicted families in the sorghum genome but also discovered 9 new miRNA candidates. Furthermore, we could correlate genotypic variation of miRNA expression with the sugar and flowering phenotypes. In addition, we found that the size distribution of small RNAs in sorghum stems was quite heterogeneous, characterized by RNAs with at least 25 nt in length that were mainly derived from ribosomal and transfer RNAs not annotated in the sorghum genome.

## Results

### Deep-sequencing of small RNAs from grain and sweet sorghum stems

**Figure 1:**
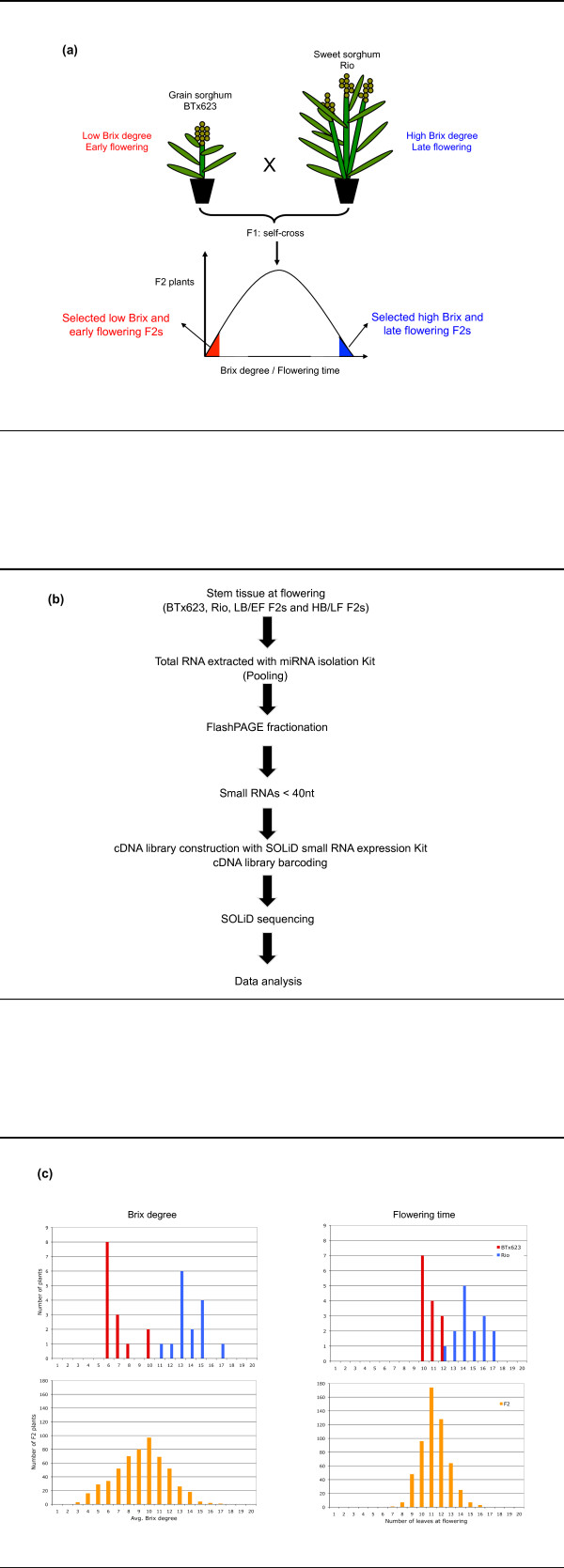
**Selection of sorghum plants and construction of stem-derived small RNA libraries for deep sequencing**. (**a**) Grain sorghum BTx623 with low Brix and early flowering phenotype, was crossed with sweet sorghum Rio with high Brix and late flowering phenotype and an F2 population was created. A total of 553 F2 plants were phenotyped for flowering time (measured as the total number of leaves at flowering) and Brix degree. Using a bulked segregant analysis (BSA) approach, we selected an equal number of F2 plants with low Brix and early flowering (LB/EF) and with high Brix and late flowering (HB/LF) phenotype, respectively. (**b**) A flow chart describing the procedure for small RNA library construction and sequencing. (**c**) Histograms displaying the Brix degree and flowering time data obtained from plants grown in the field. We selected 11 LB/EF F2s displaying Brix degree ≤ 5 and number of leaves ≤ 9, whereas the 11 HB/LF F2s selected displayed a Brix degree ≥ 13 and number of leaves ≥ 14.

We constructed five small RNAs libraries from sorghum stem tissue at the time of flowering and sequenced them using the SOLiD platform. The libraries comprised samples from BTx623, Rio, low Brix and early flowering F2 plants (LB/EF F2s), high Brix and late flowering F2 plants (HB/LF F2s), and a "mixed library" (Mix), where small RNAs from the previous four libraries were mixed in equal proportions (Figure 1).

**Figure 2:**
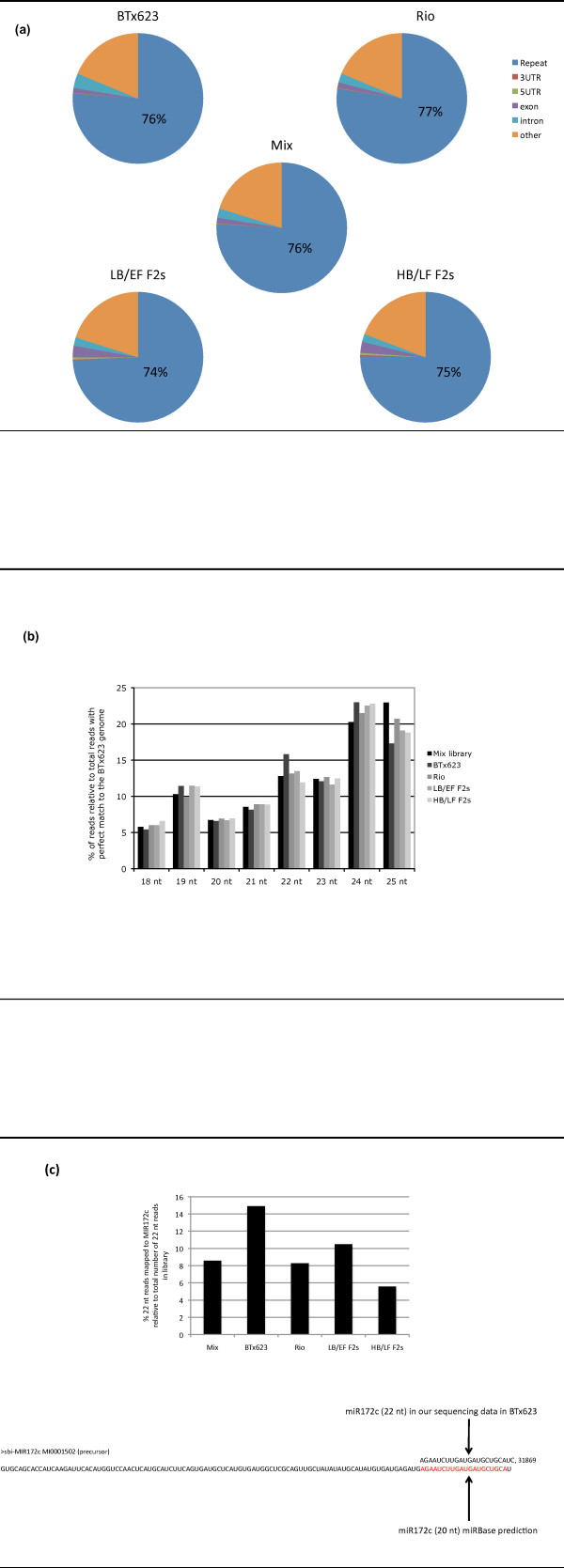
**Diversity in the small RNA content of sorghum stems**. (**a**) Mapping of small RNAs (18-25 nt) with perfect match to different elements of the BTx623 reference genome with the term "other" representing intergenic regions. (**b**) Frequency and size distribution of small RNAs reads. (**c**) A high proportion of 22 nt reads in each library are derived from miR172c locus. The small RNA reads derived from miR172c in sorghum stem tissue are 22 nt in length in contrast to the previously predicted length of 20 nt.

We obtained a total of 38,336,769 sequence reads, from which 23,008,945 (60%) matched perfectly to the BTx623 reference genome (Table 1). The reads with perfect matches that derived from repeats constituted 74 to 77% of the total reads depending on the library (Figure 2a). The non-redundant set of reads comprised 2,539,403 sequences, and the reads that were sequenced only once (termed here "singlets") comprised 2,167,946 sequences, corresponding only to 9% of the perfect matches (Table 1), suggesting that our sequencing reached a high level of saturation. If we define a cluster as two or more reads with identical sequences, the number of clusters found ranged from 20,056 in the BTx623 library to 164,623 in the HB/LF F2s library (Table 1).

**Table 1:** Deep sequencing statistics of stem-derived small RNAs

Library	# raw sequences	# perfect matches	%	# singlets	%	# clusters	Non-redundant set	%
Mix	4,023,513	2,547,108	63	276,044	11	35,083	311,127	8
BTx623	2,115,266	1,348,361	64	169,063	12	20,056	189,119	9
Rio	3,173,601	2,180,988	69	234,276	11	31,563	265,839	8
LB/EF F2s	11,974,953	7,472,940	62	653,279	9	120,132	773,411	6
HB/LF F2s	17,049,436	9,459,548	55	835,284	9	164,623	999,907	6

**Total**	38,336,769	23,008,945	60	2,167,946	9	371,457	2,539,403	8

### Diversity in the small RNA content of sorghum stems

The frequency and size distribution of small RNAs from sorghum stems revealed two interesting aspects: a peak of 25 nt small RNAs with similar abundance as the 24 nt class, and a second peak of small RNAs with 22 nt that were more abundant than the 20 and 21 nt classes, respectively (Figure 2b). This finding contrasted with the size distribution of small RNAs described for several monocot species (including small RNAs from sorghum inflorescence), in which the most abundant small RNAs were 21 and 24 nt in length, with maize being the exception, showing a larger 22 nt peak relative to the 21 nt peak [13]. This led to the hypothesis that the 22 nt class of small RNAs are specific to maize [13]. However, we have shown here that a 22 nt peak is also present in sorghum stem tissue. Furthermore, we found that a high proportion of the 22 nt reads were derived from miR172c, accounting for approximately 15% of all the 22 nt reads in the BTx623 library (Figure 2c). Our results differ from the predicted length of 20 nt for miR172c annotated in the miRBase database. Interestingly, MIR172c is located within the third intron of the Sb04g037375 gene.

The finding of small RNAs of 25 nt in length with such high abundance was unexpected. This prompted us to investigate whether they could be derived from ribosomal and/or transfer RNA genes that had not yet been annotated in the sorghum genome. Furthermore, since the sequencing read length of the SOLiD system at the time of our experiment was limited to a maximum of 25 nucleotides, it is possible that these RNAs are longer. In order to address this question, we analyzed several loci in the genome that accumulated more than thousand reads (defined as 25 nt hotspots) and found indeed that they were derived from non-annotated rRNA and tRNA genes (Additional File 1, Table S1).

In summary, we showed that the small RNA component from the stem transcriptome of sorghum is characterized by small RNAs of 22 nt in length that are mainly derived from miR172c, and by a size class of RNAs with at least 25 nt in length that are predominantly derived from rRNAs and tRNAs genes that had not been annotated in the sorghum genome.

#### Genotypic variation in the expression of known miRNAs between grain and sweet sorghum correlated with sugar content and flowering time in the F2 population

The sequencing consortium of the sorghum genome identified 149 predicted miRNAs belonging to 27 miRNA families [26], and we could detect the expression of miRNA members from 25 families based on the following criteria: a miRNA family was considered expressed only if its sequencing reads were detected in at least three libraries and with a frequency of 10 reads or more for the sum of the five libraries. A list with the reads count for each known miRNA family is provided in Additional file 2, Table S2.

**Figure 3:**
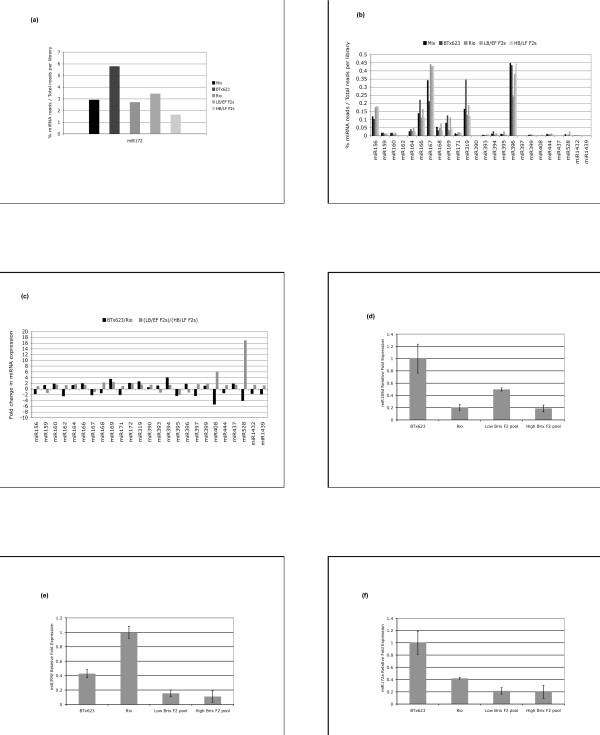
**Genotypic variations in miRNA expression**. (**a**) The miR172 is the most abundantly expressed miRNA in sorghum stems. (**b**) The rest of the known miRNAs were expressed at very low abundance (less that 0.5% of the total reads in the library) in stem tissue. (**c**) The miRNA abundances were used to calculate their relative fold change in expression between BTx623 and Rio, and between the LB/EF F2s and HB/LF F2s libraries, respectively. Positive values in the y-axis of the graph denote fold changes in miRNA expression that are higher in BTx623 relative to Rio and higher in LB/EF F2s relative to HB/LF F2s libraries, respectively; the opposite is true for negative values. The expression of miR169 and miR172 was at least twice as high in BTx623 relative to that in Rio and this difference was inherited in the F2. The opposite was true for miR395 expression. (**d-f**) Quantification of miRNA expression through Taqman Assay in pools of 10 F2 plants each with similar flowering time (10-11 leaves) but different sugar content (Brix 3-5 vs Brix 13-16), respectively. (**d**) High expression of miR169d in BTx623 relative to Rio correlates with low Brix in the F2 independently of flowering time. (**e-f**) F2 plants with similar flowering time display no differences in miR395f and miR172a expression regardless Brix degree.

The most abundantly expressed miRNA family was miR172 (Figure 3a), comprising almost 6% of the total reads with perfect match to the BTx623 genome. The rest of the known miRNAs had abundances below 0.5% (Figure 3b). When the ratio of miRNA abundances between the BTx623 and Rio libraries was compared to the ratio between the LB/EF F2s and HB/LF F2s libraries, we could identify miRNA families whose expression differences between the parents were inherited in the F2 plants (Figure 3c). Considering a cutoff level of two-fold change in miRNA expression, we found that miR169 and miR172 were expressed higher in BTx623 relative to Rio, and higher in LB/EF F2s compared to HB/LF F2s. This means that high expression of these miRNAs in BTx623 correlated with low Brix and early flowering in the F2 plants selected, and the opposite was true for miR395 (Figure 3c). Although the expression difference of miR160, miR164 and miR319 between BTx623 and Rio was inherited in the F2, and thus of interest for further analysis, it was less than two fold; so we decided to focus on miR169, miR172 and miR395 instead. The observation that high expression of miR172 correlated with early flowering is consistent with the reported role of this miRNA in the promotion of flowering [32-36].

Although miR169 and miR395 have known roles in drought stress and sulphur starvation, respectively [37,38], our data suggested a possible function for these miRNAs in sugar accumulation and flowering time. Because the pool of F2 plants used for library construction were selected based on both phenotypes, it was not possible to assign the expression inheritance pattern of both miRNAs to either sugar accumulation or flowering time alone. For this reason, additional plants from the same F2 population differing in sugar content but with similar flowering time were selected and the expression of a representative member from each miRNA family, miR169d and miR395f respectively, was quantified using the TaqMan assay. We found that high expression of miR169d in BTx623 correlated with low Brix (Figure 3d). This suggested that high expression levels of miR169 might lead to a reduction in stem sugar content regardless of flowering time. Surprisingly, high expression of miR395f in Rio relative to that in BTx623 did not correlate with sugar content in F2 plants (Figure 3e). This might indicate that high expression of miR395 would be required for flowering regardless of sugar content in the stem. Consistent with the role of miR172 in flowering, we did not observe any difference in the expression of miR172a in F2 plants with the same flowering time but different Brix (Figure 3f).

In summary, high expression of miR172 in BTx623 correlated with early flowering in the F2, whereas the opposite was true for miR395, high expression of this miRNA in Rio correlated with late flowering in the F2 plants selected. Regarding sugar content in the stem, high expression of miR169 in BTx623 correlated with low Brix in the F2 plants selected.

### Genotypic variation in the miR395/miR395* ratio

**Figure 4:**
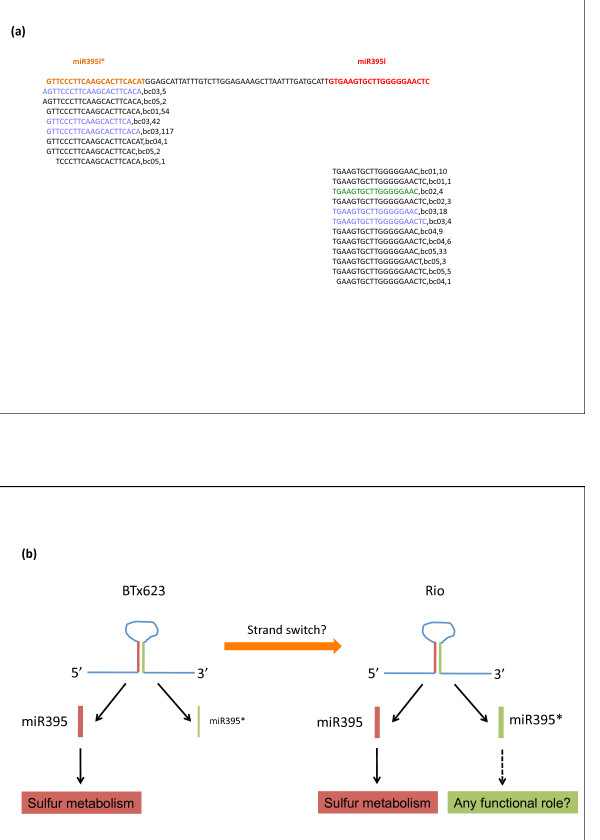
miR395* is highly abundant in Rio. (**a**) Small RNA reads derived from MIR395l are depicted. The miR395l strand sequence is shown in red whereas the miR395l* strand sequence is in orange color. In green and blue color are small RNA reads sequenced from BTx623 and Rio libraries respectively. The designation next to the small RNA reads refer to the library (bc01: Mix; bc02: BTx623; bc03: Rio; bc04: LB/EF F2s and bc05: HB/LF F2s), followed by the number of times the small RNA read was sequenced. In the BTx623 library, only reads derived from miR395l were detected whereas in the Rio library, most of the reads where derived from miR395l* instead. (**b**) Model depicting the genotypic variation in miR395/miR395* ratio where in Rio a switch towards miR395* strand production has occurred relative to BTx623. Based on miR395* high abundance in Rio, we postulate here the hypothesis that miR395* species could have a functional role in the regulation biological processes other than the sulfur metabolism previously described for miR395.

We detected the expression of the miRNA* for all *MIR395 *gene copies and this was more evident in Rio compared to BTx623, and in some instances the abundance of miR395* was even higher than that of miR395 such as the case of miR395l* for instance (Figure 4a). Indeed, when the miR395/miR395* ratio was calculated for each library, we found that miR395 reads were approximately 6 times more abundant than miR395* reads in the BTx623 library (Additional file 2, Table S2). By contrast, the abundance of miR395 relative to miR395* was in equal proportions in the Rio library. Our data highlighted a genotypic difference in the ratio between miR395 and miR395*, with a switch in strand abundance from BTx623 to Rio (Figure 4b).

### The *FRL2 *and *RR3 *genes are novel targets of miR172

Although our data might suggest a possible function of miR169 in sugar content and miR395 in flowering time, we could not detect any predicted target related to carbohydrate metabolism and flowering time respectively (Additional file 3, Table S3 and Additional file 4, Figure S1). Thus, the expression of miR169 and miR395 target genes, and their correlation with Brix and flowering phenotypes remains to be elucidated. Regarding the miR172-predicted targets, we detected cleavage products for the genes *INDETERMINATE SPIKELET 1 (IDS1) *and an AP2 transcription factor (Additional file 3, Table S3; Additional file 4, Figure S1; and Additional file 5, Figure S2). Furthermore, when the expression of these two miR172 target genes was tested, we found that they were expressed higher in Rio compared with BTx623 as expected. However, we could not find a correlation between their expression levels with the flowering phenotype in the F2 pools of plants selected (data not shown).

A *FRIGIDA-like 2 *(*FRL2*) and a *TYPE A RESPONSE REGULATOR 3 *(*RR3*) were predicted as new targets of miR172 with the cleavage product of *FRL2 *experimentally validated in this study (Additional file 5, Figure S2). The *FRIGIDA*-related genes are a major determinant of natural variation in the winter-annual habit between *Arabidopsis *accessions [39,40], whereas the *TYPE A RESPONSE REGULATOR 3 *(*ARR3*) has a function in the circadian clock [41]. Although sorghum is a crop from semi-arid regions [26], the miR172-mediated post-transcriptional regulation of *FRL2 *might have a role in the adaptation of sorghum to temperate climates. Consistent with this, a role of miR172 in the regulation of flowering time by ambient temperature in *Arabidopsis *has been recently described [42].

### Identification of new miRNAs

**Figure 5:**
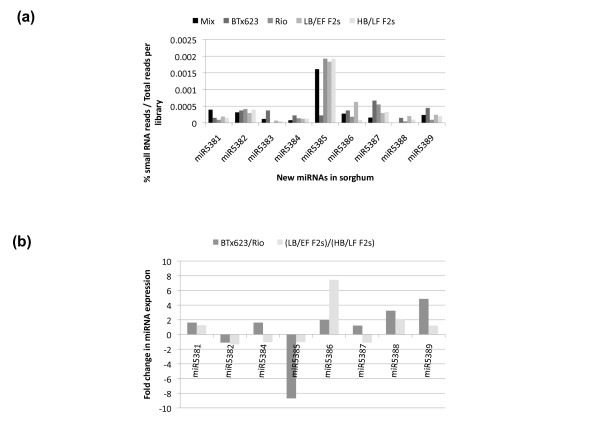
**Genotypic variations in the expression of new miRNAs**. (**a**) The frequency count of small RNAs for each new miRNA was used to calculate its abundance. (**b**) The miRNA abundances were used to calculate their relative fold change in expression between BTx623 and Rio, and between the LB/EF F2s and HB/LF F2s libraries, respectively. Positive values in the y-axis of the graph denote fold changes in miRNA expression that are higher in BTx623 relative to Rio and higher in LB/EF F2s relative to HB/LF F2s libraries, respectively; the opposite is true for negative values. The miRNA miR5383 was not included in the graph because it was not detected in the Rio library (see Additional file 9, Table S4).

The miRDeep pipeline [43] was adapted for *de novo *detection of miRNAs in sorghum (Additional file 6, Figure S3). From an original set of 223 predicted hairpins in the sorghum genome, 9 met the miRNA annotation criteria previously established [44], (Table 2 and Additional file 7, Figure S4). All the new miRNAs have predicted genes as targets except miR5389 (Additional file 8, Figure S5). All predicted 9 miRNAs met the expression criteria used above for known miRNAs (Figure 5 and Additional file 9, Table S4). From all miRNAs whose expression could be detected in sorghum stems, two of them were found to be within introns of protein coding genes, these included miR172c and miR437g.

**Table 2:** List of new predicted MIR genes in the sorghum genome

MIR gene ID	Position	Strand	miRNA size	miRNA sequence 5'-3'	miRNA* sequence 5'-3'	miRNA* size
sbi-MIR5381	Ch1: 574388..574497	+	19	AAGATCTGTGGCGCCGAGC	TCGGCGCTAAGATCTCTGG	19
sbi-MIR5382	Ch2: 1930828..1930937	+	18	CCAATCTAAACAGGCCCT	GACCTGTTTAGATTGGGA	18
sbi-MIR5383	Ch4: 43242765..43242874	+	24	ATGACAGAGCTCCGGCAGAGATAT	TTCTCCGCCGAGCTTATCTGTGG	23
sbi-MIR5384	Ch4: 45785396..45785505	+	18	CGCGCCGCCGTCCAGCGG	CTTGGCCGGTGCACGCGTC	19
sbi-MIR5385	Ch6: 56307517..56307626	+	22	ACCACCAACCCCACCGCTTCTC	GAAGCGGTGGTGTTGGTGGTGA	22
sbi-MIR5386	Ch7: 877244..877353	+	20	CGTCGCTGTCGCGCGCGCTG	GGTCAGGGCAGAGCACGCA	19
sbi-MIR5387	Ch7: 15969322..15969431	+	25	TAACACGAACCGGTGCTAAAGGATC	CCCTTTAGCACCGGTTCGTGTTACA	25
sbi-MIR5388	Ch8: 1629110..1629219	+	22	ATCTTTGCCGGGTGTCTCTGAC	CAGCAAACATTCGGCAAAGAAAA	23
sbi-MIR5389	Ch8: 4848342..4848451	+	21	GCTTGAGTTTATCAGCCGAGT	ATGGCTTATCAGCCAAGTGA	20

*All the small RNA reads mapped to "chromosome_7_22.BC_03" were derived from the predicted miRNA* strand

From the newly identified miRNAs, miR5386, and miR5388 displayed allelic variation in expression between BTx623 and Rio that was inherited in the F2 offspring (Figure 5). However, the predicted target genes for miR5386 did not include any transcript involved neither in flowering nor in carbohydrate metabolism. This was a similar case with miR5388, with no predicted targets involved in flowering but with two genes involved in carbohydrate metabolism as predicted targets, encoding the beta subunit 1 and 2 of the Snf-1 related protein kinase (SnRK1) respectively [45] (Additional file 8, Figure S5).

We next attempted to experimentally validate the miRNA-mediated cleavage of predicted targets. Potential miRNA target sites were scored from 0 to 8 (see Methods), with higher scores indicating less confidence in the predictions. We tested 14 predicted targets with scores less than 4 but we could not detect the miRNA-mediated cleavage for any of them. A low rate in target validation has also been observed for newly predicted miRNAs in tomato, with three targets validated from 65 predicted targets that were tested [46]. Recently, a similar case of very low rate in target validation was reported for predicted targets of new miRNAs identified in *Arabidopsis lyrata *[47].

## Discussion

Here we have described the first characterization of the small RNA component of the transcriptome from sorghum stems. The choice of stems as plant material is interesting not only because it is the tissue were fermentable sugars do accumulate, but it is also the venue for the movement of small RNA duplexes (siRNAs and miRNAs) from source to sink tissues, as have been recently demonstrated [48,49]. Thus, one could expect the small RNA component of the stem to be quite diverse or heterogeneous. Indeed, the unexpected finding of a high abundance peak of RNAs with 25 nt or more in length lead us to the finding of rRNA and tRNA genes that have not been annotated yet in the sorghum genome. We have also shown that the abundance of the 22 nt small RNAs in sorghum stem tissue was greater than the 20 and 21 nt small RNAs respectively. Our results contrast the recently proposed notion that the 22 nt peak of small RNAs is exclusive of maize [13]. Furthermore, we found that up to 15% of all the 22 nt small RNAs in the BTx623 library were derived from miR172c, which has been previously predicted to have a length of 20 nt (Paterson et al. 2009). Recently, 22 nt miRNAs have been described to trigger siRNA biogenesis from target transcripts in *Arabidopsis *[50,51]. Thus, it would be interesting to test if miR172c can also trigger siRNA biogenesis in sorghum.

As expected, the specific genetic material, tissue sample and developmental stage used in our study, allowed us to capture a broad spectrum of the small RNA component of the sorghum transcriptome. On the other hand, the specificity of the material also permitted us to gain new insights into how complex traits like sugar accumulation and flowering time might be regulated at the post-transcriptional level. Such regulation of gene expression could provide an opportunity to manipulate biofuel traits, where stem sugar rather than cellulose and increased biomass because of delayed flowering could be enhanced [52]. By taking a genetic approach in conjunction with deep-sequencing of stem-derived small RNAs, we were able to correlate variation in miRNA expression between grain and sweet sorghum, with the sugar and flowering phenotypes of selected F2 plants derived from their cross. However, analysis of the differential accumulation of potential target genes did not exhibit a simple correlation with miRNA levels. Therefore, further studies will be required to unveil the underlying mechanisms between genotype and phenotype.

In the case of miR395, it is interesting to note that there was genotypic variation in the miR395/miR395* ratio, with the Rio genotype expressing both strands at equal proportions in contrast to a clear predominance of miR395 abundance over miR395* in BTx623 (Figure 4b). This is reminiscent of the recently proposed "arm switching" model of miRNA evolution described for nematodes species [53], in which the mature miRNA is produced from the 5' arm of the miRNA hairpin in a particular species but in a different nematode species the 5' arm of the same MIR gene gives rise to the miRNA* instead. Interestingly, it has been shown recently that miRNA* species have physiological relevance in *Drosophila*, since a significant number of them are well conserved, can be loaded into the RISC complex through their preferential association with ARGONAUTE2 (AGO2) rather that AGO1, and can also regulate the expression of target genes [54]. Furthermore, the regulatory potential of miRNA* species in vertebrates has been recently demonstrated as well [55].

## Conclusions

Based on the above, several interesting questions can be formulated. First, does miR395* have any regulatory potential? Second, what is the mechanism behind the genotypic difference in miR395/miR395* ratio? Third, is this ratio altered in a developmental and/or tissue dependent manner? Fourth, is this an example of a general phenomenon? If this is the case, we would envision that other miRNAs families as well will display differences in their miRNA/miRNA* ratio dependent on the genotype and/or condition. Future work will be required to provide a better understanding of miR395's involvement in processes other than its previously described role in sulfur metabolism.

## Methods

### Plant material

The grain (BTx623) and sweet (Rio) sorghum cultivars together with F2 plants derived from their cross were grown in the field of the Waksman Institute during the summer of 2008. The juice from three internodes of the main stem was harvested at the time of flowering and the Brix degree measured as previously described [30,31]. The average Brix degree from three internodes per plant was used. Flowering time was measured as the number of leaves in the main stem at the time of anthesis.

In total, 15 plants for each parent and 553 F2 plants were scored for Brix degree and flowering time. The F2 plants selected for sequencing had either low Brix (Brix ≤ 5)/early flowering (N0 leaves ≤ 9) or high Brix (Brix ≥ 13)/late flowering (NO leaves ≥ 14).

### Construction of small RNA libraries

Total RNA from internode tissue was extracted at the time of flowering with the mirVana miRNA isolation kit (Ambion). RNA extraction was performed in 5 independent plants for each BTx623 and Rio, and 11 independent plants for each low Brix/early flowering and high Brix/late flowering F2 plants respectively. The total RNA (1 μg per sample) was pooled and then fractionated with the flashPage fractionator (Ambion) to isolate RNAs smaller that 40 nt in length. The isolated small RNAs were used to construct small RNA cDNA libraries with the SOLiD small RNA library construction kit (Ambion). The sequencing was carried out at the Waksman genomics laboratory on the SOLiD 3 platform, which has a read length limit of 25 nt http://solid.rutgers.edu.

### Bioinformatic analysis

We mapped the 25 nt long reads to the sorghum genome using the SHRiMP program version 1.0.5 [56], with default parameter settings except that the number of matches was limited to 10. SHRiMP allowed us to perform the alignments in SOLiD's colorspace. For the further analyses we used only alignments that matched perfectly to the genome starting from the first position in the read up to the sequencing primer. Because the SOLiD 3 platform had a read length limit of 25 nucleotides, adaptor sequences did not have to be trimmed prior mapping to the genome. As a consequence, we could estimate the length of an individual sequence read by one base with a probability of 0.25. These reads were then clustered with Vmatch http://vmatch.de/ to reduce the number of identical reads for downstream analyses. We required 100% identity among the sequences of a cluster. We have further filtered the clustered reads against the repetitive elements of sorghum and used the remaining sequences for *de novo *prediction of miRNA using miRDeep.

We defined a 25 nt "hotspot" as those loci in the genome that displayed a high coverage of 25 nt reads, in our case thousand reads. The length of the hotspot was determined as each consecutive interrogated base that had more than thousand 25 nt reads mapped to it.

### Quantification of miRNA expression

The TaqMan MicroRNA Assays (Applied Biosystems) was used to quantify the expression of miR172a, and the Custom TaqMan Small RNA Assays (Applied Biosystems) was used to quantify the expression of miR169d and miR395f respectively. The qRT-PCR reaction was done using the MyiQ Real-Time PCR Detection System (BIO-RAD Laboratories, Inc.). A relative quantification normalized against unit mass (10 ng total RNA) was performed as previously described [30].

### *De novo *discovery of sorghum miRNAs

For *de novo *prediction of potential miRNAs, we have used the miRDeep package [43]. As miRDeep does not take colorspace alignment as input, we had to reshape the output to miRDeep's blastparse format. Moreover, the SHRiMP alignment scores and the score used had to be recalculated to fit miRDeep's blastparse format. We used the same formula and method as described [57]. At this point, we also had to translate the color space two base encoding sequences into standard nucleotide base space sequences. As we considered only perfectly matching reads after the initial alignment to the genome, we could easily translate from color space to base space sequence format. The subsequent de novo calling of miRNAs was carried out as described [43,57].

Finally, the coordinates of de novo miRNAs that were predicted on the minus strand were corrected as miRDeep refers the coordinates to the 5' end of the minus strand. Though, conventionally the coordinates refer always to the 5' end of the plus strand. The GenBank accession numbers for the new miRNAs are sbi-MIR538.sqn sbi-MIR5381 JN205291; sbi-MIR538.sqn sbi-MIR5382 JN205292; sbi-MIR538.sqn sbi-MIR5383 JN205293; sbi-MIR538.sqn sbi-MIR5384 JN205294; sbi-MIR538.sqn sbi-MIR5385 JN205295; sbi-MIR538.sqn sbi-MIR5386 JN205296; sbi-MIR538.sqn sbi-MIR5387 JN205297; sbi-MIR538.sqn sbi-MIR5388 JN205298; sbi-MIR538.sqn sbi-MIR5389 JN205299.

We have also validated all potential new miRNAs according to the annotation criteria proposed by [44].

### Target prediction and validation

We have used the novel miRNAs for a target prediction. Firstly, we compared the sequences to the unspliced transcripts of sorghum [26], with BLASTN using these parameters: -F F -W 7 -e 1 -q -2 -G -1. We scored each base of the alignment according to these criteria: match as 0; GU pairs as 0.5; gaps as 2; all other pairs were scored as 1. We doubled the score within the first 13 bases of the miRNA/alignment. We considered the gene as a potential target if the total score of the alignment was equal to or less than 8.

The miRNA-mediated cleavage of mRNAs was performed through a modified procedure of the RLM-RACE protocol from Invitrogen. The sequences of the reverse primers used in the modified RACE are: Sb01g044240 (5' GCCCATATGGACGGAAGATA 3'); Sb02g007000 (5' CTGGTAGCCGGAGAACAACT 3') and Sb06g030670 (5' TTTCATCAGTGCTTGCCAAT 3'). The validation of predicted targets was performed in BTx623 or Rio cultivars only. Annotation of the miRNA gene targets was based on the Phytozome database http://www.phytozome.net.

## Authors' contributions

MC, RB, and JM designed the study and wrote the manuscript. MC carried out the experimental work and RB the computational work. All authors read and approved the final version of the manuscript.

## Supplementary Material

Additional File 1 **Table S1 - 25 nt hotspots in the sorghum genome**. Click here for file

Additional file 2 **Frequency counts of small RNA reads for known microRNA families**. Table S2 displays a quantitative analysis of microRNAs. Click here for file

Additional file 3 **Predicted targets of miR169, miR172, and miR395**. Table S3 provides a list of predicted target genes of miR169, miR172, and miR395. Click here for file

Additional file 4 **Targets of predicted for miR169, miR172 and miR395 microRNAs**. Figure S1 displays an alignment between miR169, miR172 and miR395 microRNAs and their target sequences. Click here for file

Additional file 5 **Mapping of miR172-guided cleavage sites in predicted target genes**. Figure S2 displays an alignment of miR172 with its target sequences and cleavage sites. The locations of the miRNA-cleavage sites are indicated with downward arrows and the frequency of the cleavages are indicated as the number of clones for each RACE product with respect to the total clones sequenced. Click here for file

Additional file 6 **Pipeline for the *de novo *miRNA detection**. Figure S3 presents a diagram of computational steps involved in de novo miRNA detection. All reads from SOLiD sequencing were mapped in colorspace to the sorghum genome using SHRiMP. Perfect matching reads were clustered with Vmatch then filtered against the sorghum repeat sequences and compared with know sorghum miRNAs to classify them. The remaining sequences were taken for *de novo *miRNA prediction using miRDeep. Click here for file

Additional file 7 **Hairpin structures of the newly discovered miRNAs**. Figure S4 presents a collection of hairpin structures from newly discovered miRNAs. Sequences are depicted together with the frequency distribution of the small RNA reads aligned to the hairpin. The 2D hairpin structure produced by the miRDeep software is also shown. Click here for file

Additional file 8 **Predicted targets for the newly discovered miRNAs in sorghum**. Figure S5 presents a list of alignments between new discovered miRNAs and their predicted targets in sorghum. Click here for file

Additional file 9 **Frequency counts of small RNA reads for new MIR genes**. Table S4 displays a quantitative analysis of new MIR genes. Click here for file

## References

[B1] Chuck G Candela H Hake S Big impacts by small RNAs in plant development Current Opinion in Plant Biology 2009 12 1 81 86 10.1016/j.pbi.2008.09.008 18980858

[B2] Vaucheret H Post-transcriptional small RNA pathways in plants: mechanisms and regulations Genes Dev 2006 20 7 759 771 10.1101/gad.1410506 16600909

[B3] Zamore PD Haley B Ribo-gnome: the big world of small RNAs Science 2005 309 5740 1519 1524 10.1126/science.1111444 16141061

[B4] Bartel DP MicroRNAs: genomics, biogenesis, mechanism, and function Cell 2004 116 2 281 297 10.1016/S0092-8674(04)00045-5 14744438

[B5] Vazquez F Arabidopsis endogenous small RNAs: highways and byways Trends in Plant Science 2006 11 9 460 468 10.1016/j.tplants.2006.07.006 16893673

[B6] Lee Y Kim M Han J Yeom KH Lee S Baek SH Kim VN MicroRNA genes are transcribed by RNA polymerase II EMBO J 2004 23 20 4051 4060 10.1038/sj.emboj.7600385 15372072 PMC524334

[B7] Henderson IR Zhang X Lu C Johnson L Meyers BC Green PJ Jacobsen SE Dissecting Arabidopsis thaliana DICER function in small RNA processing, gene silencing and DNA methylation patterning Nat Genet 2006 38 6 721 725 10.1038/ng1804 16699516

[B8] Filipowicz W Bhattacharyya SN Sonenberg N Mechanisms of post-transcriptional regulation by microRNAs: are the answers in sight? Nat Rev Genet 2008 9 2 102 114 18197166 10.1038/nrg2290

[B9] Khraiwesh B Arif MA Seumel GI Ossowski S Weigel D Reski R Frank W Transcriptional control of gene expression by microRNAs Cell 2010 140 1 111 122 10.1016/j.cell.2009.12.023 20085706

[B10] Wu L Zhou H Zhang Q Zhang J Ni F Liu C Qi Y DNA methylation mediated by a microRNA pathway Mol Cell 2010 38 3 465 475 10.1016/j.molcel.2010.03.008 20381393

[B11] Lu C Tej SS Luo S Haudenschild CD Meyers BC Green PJ Elucidation of the small RNA component of the transcriptome Science 2005 309 5740 1567 1569 10.1126/science.1114112 16141074

[B12] Nobuta K Venu RC Lu C Beló A Vemaraju K Kulkarni K Wang W Pillay M Green PJ Wang GL Meyers BC An expression atlas of rice mRNAs and small RNAs Nat Biotechnol 2007 25 4 473 477 10.1038/nbt1291 17351617

[B13] Nobuta K Lu C Shrivastava R Pillay M De Paoli E Accerbi M Arteaga-Vazquez M Sidorenko L Jeong DH Yen Y Green PJ Chandler VL Meyers BC Distinct size distribution of endogeneous siRNAs in maize: Evidence from deep sequencing in the mop1-1 mutant Proc Natl Acad Sci USA 2008 105 39 14958 14963 10.1073/pnas.0808066105 18815367 PMC2567475

[B14] Wang X Elling AA Li X Li N Peng Z He G Sun H Qi Y Liu XS Deng XW Genome-wide and organ-specific landscapes of epigenetic modifications and their relationships to mRNA and small RNA transcriptomes in maize Plant Cell 2009 21 4 1053 1069 10.1105/tpc.109.065714 19376930 PMC2685623

[B15] Wei B Cai T Zhang R Li A Huo N Li S Gu YQ Vogel J Jia J Qi Y Mao L Novel microRNAs uncovered by deep sequencing of small RNA transcriptomes in bread wheat (Triticum aestivum L.) and Brachypodium distachyon (L.) Beauv Funct Integr Genomics 2009 9 4 499 511 10.1007/s10142-009-0128-9 19499258

[B16] Heisel SE Zhang Y Allen E Guo L Reynolds TL Yang X Kovalic D Roberts JK Characterization of unique small RNA populations from rice grain PLoS ONE 2008 3 8 e2871 10.1371/journal.pone.0002871 18716673 PMC2518513

[B17] Sunkar R Girke T Jain PK Zhu JK Cloning and characterization of microRNAs from rice Plant Cell 2005 17 5 1397 1411 10.1105/tpc.105.031682 15805478 PMC1091763

[B18] Sunkar R Zhou X Zheng Y Zhang W Zhu JK Identification of novel and candidate miRNAs in rice by high throughput sequencing BMC Plant Biol 2008 8 25 10.1186/1471-2229-8-25 18312648 PMC2292181

[B19] Xue LJ Zhang JJ Xue HW Characterization and expression profiles of miRNAs in rice seeds Nucleic Acids Res 2009 37 3 916 930 10.1093/nar/gkn998 19103661 PMC2647296

[B20] Zhu QH Spriggs A Matthew L Fan L Kennedy G Gubler F Helliwell C A diverse set of microRNAs and microRNA-like small RNAs in developing rice grains Genome Res 2008 18 9 1456 1465 10.1101/gr.075572.107 18687877 PMC2527712

[B21] Jannoo N Grivet L Chantret N Garsmeur O Glaszmann JC Arruda P D'Hont A Orthologous comparison in a gene-rich region among grasses reveals stability in the sugarcane polyploid genome Plant J 2007 50 4 574 585 10.1111/j.1365-313X.2007.03082.x 17425713

[B22] Glasziou K Gayler R Storage of sugars in stalks of sugarcane Bot Rev 1972 38 471 490 10.1007/BF02859248

[B23] Hoffman-Thoma G Hinkel K Nicolay P Willenbrink J Sucrose accumulation in sweet sorghum stem internodes in relation to growth Physiologia Plantarum 1996 97 277 284 10.1034/j.1399-3054.1996.970210.x

[B24] Goldemberg J Ethanol for a sustainable energy future Science 2007 315 808 810 10.1126/science.1137013 17289989

[B25] Grivet L Arruda P Sugarcane genomics: depicting the complex genome of an important tropical crop Curr Opin Plant Biol 2002 5 122 127 10.1016/S1369-5266(02)00234-0 11856607

[B26] Paterson AH Bowers JE Bruggmann R Dubchak I Grimwood J Gundlach H Haberer G Hellsten U Mitros T Poliakov A Schmutz J Spannagl M Tang H Wang X Wicker T Bharti AK Chapman J Feltus FA Gowik U Grigoriev IV Lyons E Maher CA Martis M Narechania A Otillar RP Penning BW Salamov AA Wang Y Zhang L Carpita NC The Sorghum bicolor genome and the diversification of grasses Nature 2009 457 7229 551 556 10.1038/nature07723 19189423

[B27] Ritter KB McIntyre CL Godwin ID Jordan DR Chapman SC An assesment of the genetic relationship between sweet and grain sorghums within Sorghum bicolor ssp. bicolor (L.) Moench using AFLP markers Euphytica 2007 157 161 176 10.1007/s10681-007-9408-4

[B28] Murray S Sharma A Rooney W Klein P Mullet J Mitchell S Kresovich S Genetic Improvement of Sorghum as a Biofuel Feedstock: I. QTL for Stem Sugar and Grain Nonstructural Carbohydrates Crop Science 2008 48 6 2165 2179 10.2135/cropsci2008.01.0016

[B29] Ritter KB Jordan DR Chapman SC Godwin ID Mace E McIntyre CL Identification of QTL for sugar-related traits in a sweet × grain sorghum (Sorghum bicolor L. Moench) recombinant inbred population Molecular Breeding 2008 22 367 384 10.1007/s11032-008-9182-6

[B30] Calviño M Bruggmann R Messing J Screen of genes linked to high sugar content in stems by comparative genomics Rice 2008 1 166 176 10.1007/s12284-008-9012-9

[B31] Calviño M Miclaus M Bruggmann R Messing J Molecular markers for sweet sorghum based on microarray expression data Rice 2009 2 2 129 142 10.1007/s12284-009-9029-8

[B32] Chuck G Meeley R Irish E Sakai H Hake S The maize tasselseed4 microRNA controls sex determination and meristem cell fate by targeting Tasselseed6/indeterminate spikelet1 Nat Genet 2007 39 12 1517 1521 10.1038/ng.2007.20 18026103

[B33] Lauter N Kampani A Carlson S Goebel M Moose SP microRNA172 down-regulates glossy15 to promote vegetative phase change in maize Proc Natl Acad Sci USA 2005 102 26 9412 9417 10.1073/pnas.0503927102 15958531 PMC1166634

[B34] Mathieu J Yant LJ Mürdter F Küttner F Schmid M Repression of flowering by the miR172 target SMZ PLoS Biol 2009 7 7 e1000148 10.1371/journal.pbio.1000148 19582143 PMC2701598

[B35] Wu G Park MY Conway SR Wang JW Weigel D Poethig RS The sequential action of miR156 and miR172 regulates developmental timing in Arabidopsis Cell 2009 138 4 750 759 10.1016/j.cell.2009.06.031 19703400 PMC2732587

[B36] Zhu QH Upadhyaya NM Gubler F Helliwell CA Over-expression of miR172 causes loss of spikelet determinacy and floral organ abnormalities in rice (Oryza sativa) BMC Plant Biol 2009 9 1 149 10.1186/1471-2229-9-149 20017947 PMC2803185

[B37] Kawashima CG Yoshimoto N Maruyama-Nakashita A Tsuchiya YN Saito K Takahashi H Dalmay T Sulphur starvation induces the expression of microRNA-395 and one of its target genes but in different cell types Plant J 2009 57 2 313 321 10.1111/j.1365-313X.2008.03690.x 18801012

[B38] Li WX Oono Y Zhu J He XJ Wu JM Iida K Lu XY Cui X Jin H Zhu JK The Arabidopsis NFYA5 transcription factor is regulated transcriptionally and posttranscriptionally to promote drought resistance Plant Cell 2008 20 8 2238 2251 10.1105/tpc.108.059444 18682547 PMC2553615

[B39] Michaels SD Bezerra IC Amasino RM FRIGIDA-related genes are required for the winter-annual habit in Arabidopsis Proc Natl Acad Sci USA 2004 101 9 3281 3285 10.1073/pnas.0306778101 14973192 PMC365781

[B40] Schläppi MR FRIGIDA LIKE 2 is a functional allele in Landsberg erecta and compensates for a nonsense allele of FRIGIDA LIKE 1 Plant Physiology 2006 142 4 1728 1738 10.1104/pp.106.085571 17056759 PMC1676065

[B41] Salomé PA To JP Kieber JJ McClung CR Arabidopsis response regulators ARR3 and ARR4 play cytokinin-independent roles in the control of circadian period Plant Cell 2006 18 1 55 69 10.1105/tpc.105.037994 16326927 PMC1323484

[B42] Lee H Yoo SJ Lee JH Kim W Yoo SK Fitzgerald H Carrington JC Ahn JH Genetic framework for flowering-time regulation by ambient temperature-responsive miRNAs in Arabidopsis Nucleic Acids Res 2010 38 3081 3093 10.1093/nar/gkp1240 20110261 PMC2875011

[B43] Friedländer MR Chen W Adamidi C Maaskola J Einspanier R Knespel S Rajewsky N Discovering microRNAs from deep sequencing data using miRDeep Nat Biotechnol 2008 26 4 407 415 10.1038/nbt1394 18392026

[B44] Meyers BC Axtell MJ Bartel B Bartel DP Baulcombe D Bowman JL Cao X Carrington JC Chen X Green PJ Griffiths-Jones S Jacobsen SE Mallory AC Martienssen RA Poethig RS Qi Y Vaucheret H Voinnet O Watanabe Y Weigel D Zhu JK Criteria for annotation of plant MicroRNAs Plant Cell 2008 20 12 3186 3190 10.1105/tpc.108.064311 19074682 PMC2630443

[B45] Zheng Z Xu X Crosley RA Greenwalt SA Sun Y Blakeslee B Wang L Ni W Sopko MS Yao C Yau K Burton S Zhuang M McCaskill DG Gachotte D Thompson M Green TW The protein kinase SnRK2.6 mediates the regulation of sucrose metabolism and plant growth in Arabidopsis Plant Physiology 2010 153 1 99 113 10.1104/pp.109.150789 20200070 PMC2862418

[B46] Moxon S Jing R Szittya G Schwach F Rusholme RL Pilcher Moulton V Dalmay T Deep sequencing of tomato short RNAs identifies microRNAs targeting genes involved in fruit ripening Genome Res 2008 18 10 1602 1609 10.1101/gr.080127.108 18653800 PMC2556272

[B47] Ma Z Coruh C Axtell MJ Arabidopsis lyrata small RNAs: transient MIRNA and small interfering RNA loci within the Arabidopsis genus Plant Cell 2010 22 4 1090 1103 10.1105/tpc.110.073882 20407023 PMC2879747

[B48] Dunoyer P Schott G Himber C Meyer D Takeda A Carrington JC Voinnet O Small RNA duplexes function as mobile silencing signals between plant cells Science 2010 328 5980 912 916 10.1126/science.1185880 20413458

[B49] Molnar A Melnyk CW Bassett A Hardcastle TJ Dunn R Baulcombe DC Small silencing RNAs in plants are mobile and direct epigenetic modification in recipient cells Science 2010 328 5980 872 875 10.1126/science.1187959 20413459

[B50] Chen HM Chen LT Patel K Li YH Baulcombe DC Wu SH 22-Nucleotide RNAs trigger secondary siRNA biogenesis in plants Proc Natl Acad Sci USA 2010 107 34 15269 15274 10.1073/pnas.1001738107 20643946 PMC2930544

[B51] Cuperus JT Carbonell A Fahlgren N Garcia-Ruiz H Burke RT Takeda A Sullivan CM Gilbert SD Montgomery TA Carrington JC Unique functionality of 22-nt miRNAs in triggering RDR6-dependent siRNA biogenesis from target transcripts in Arabidopsis Nat Struct Mol Biol 2010 17 8 997 1003 10.1038/nsmb.1866 20562854 PMC2916640

[B52] Torney F Moeller L Scarpa A Wang K Genetic engineering approaches to improve bioethanol production from maize Current Opinion in Biotechnology 2007 18 3 193 199 10.1016/j.copbio.2007.03.006 17399975

[B53] de Wit E Linsen SEV Cuppen E Berezikov E Repertoire and evolution of miRNA genes in four divergent nematode species Genome Res 2009 19 11 2064 2074 10.1101/gr.093781.109 19755563 PMC2775598

[B54] Ghildiyal M Xu J Seitz H Weng Z Zamore PD Sorting of Drosophila small silencing RNAs partitions microRNA* strands into the RNA interference pathway RNA 2010 16 43 56 10.1261/rna.1972910 19917635 PMC2802036

[B55] Yang JS Phillips MD Betel D Mu P Ventura A Siepel AC Chen KC Lai EC Widespread regulatory activity of vertebrate microRNA* species RNA 2011 17 2 312 326 10.1261/rna.2537911 21177881 PMC3022280

[B56] Rumble SM Lacroute P Dalca AV Fiume M Sidow A Brudno M SHRiMP: accurate mapping of short color-space reads PLoS Comput Biol 2009 5 5 e1000386 10.1371/journal.pcbi.1000386 19461883 PMC2678294

[B57] Goff LA Davila J Swerdel MR Moore JC Cohen RI Wu H Sun YE Hart RP Ago2 immunoprecipitation identifies predicted microRNAs in human embryonic stem cells and neural precursors PLoS ONE 2009 4 9 e7192 10.1371/journal.pone.0007192 19784364 PMC2745660

