# Supplementary figures and images for: Characterization of the small RNA component of the transcriptome from grain and sweet sorghum stems
Source: BMC Genomics. 2011 Jul 8;12:356. doi: 10.1186/1471-2164-12-356 (PMC3143107; doi:10.1186/1471-2164-12-356)

## Slide 1
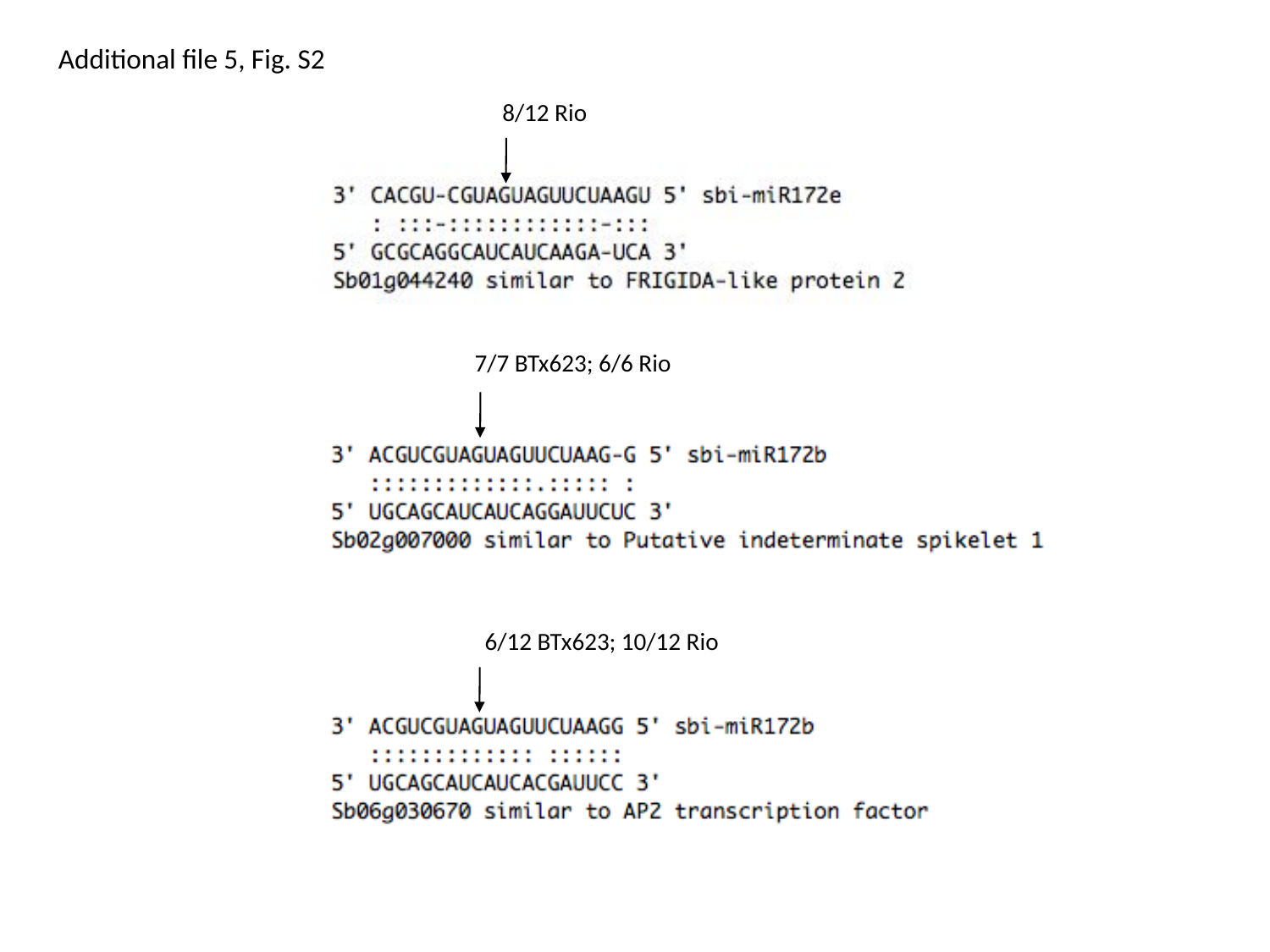

Additional file 5, Fig. S2
8/12 Rio
7/7 BTx623; 6/6 Rio
6/12 BTx623; 10/12 Rio

Supplement: Additional file 5 — Mapping of miR172-guided cleavage sites in predicted target genes. Figure S2 displays an alignment of miR172 with its target sequences and cleavage sites. The locations of the miRNA-cleavage sites are indicated with downward arrows and the frequency of the cleavages are indicated as the number of clones for each RACE product with respect to the total clones sequenced. [file 1471-2164-12-356-S5.PPT]
