# Supplementary material for: Characterization of the small RNA component of the transcriptome from grain and sweet sorghum stems
Source: BMC Genomics. 2011 Jul 8;12:356. doi: 10.1186/1471-2164-12-356 (PMC3143107; doi:10.1186/1471-2164-12-356)
Supplement: Additional file 6 — Pipeline for the de novo miRNA detection. Figure S3 presents a diagram of computational steps involved in de novo miRNA detection. All reads from SOLiD sequencing were mapped in colorspace to the sorghum genome using SHRiMP. Perfect matching reads were clustered with Vmatch then filtered against the sorghum repeat sequences and compared with know sorghum miRNAs to classify them. The remaining sequences were taken for de novo miRNA prediction using miRDeep. [file 1471-2164-12-356-S6.PPT]

## Slide 1
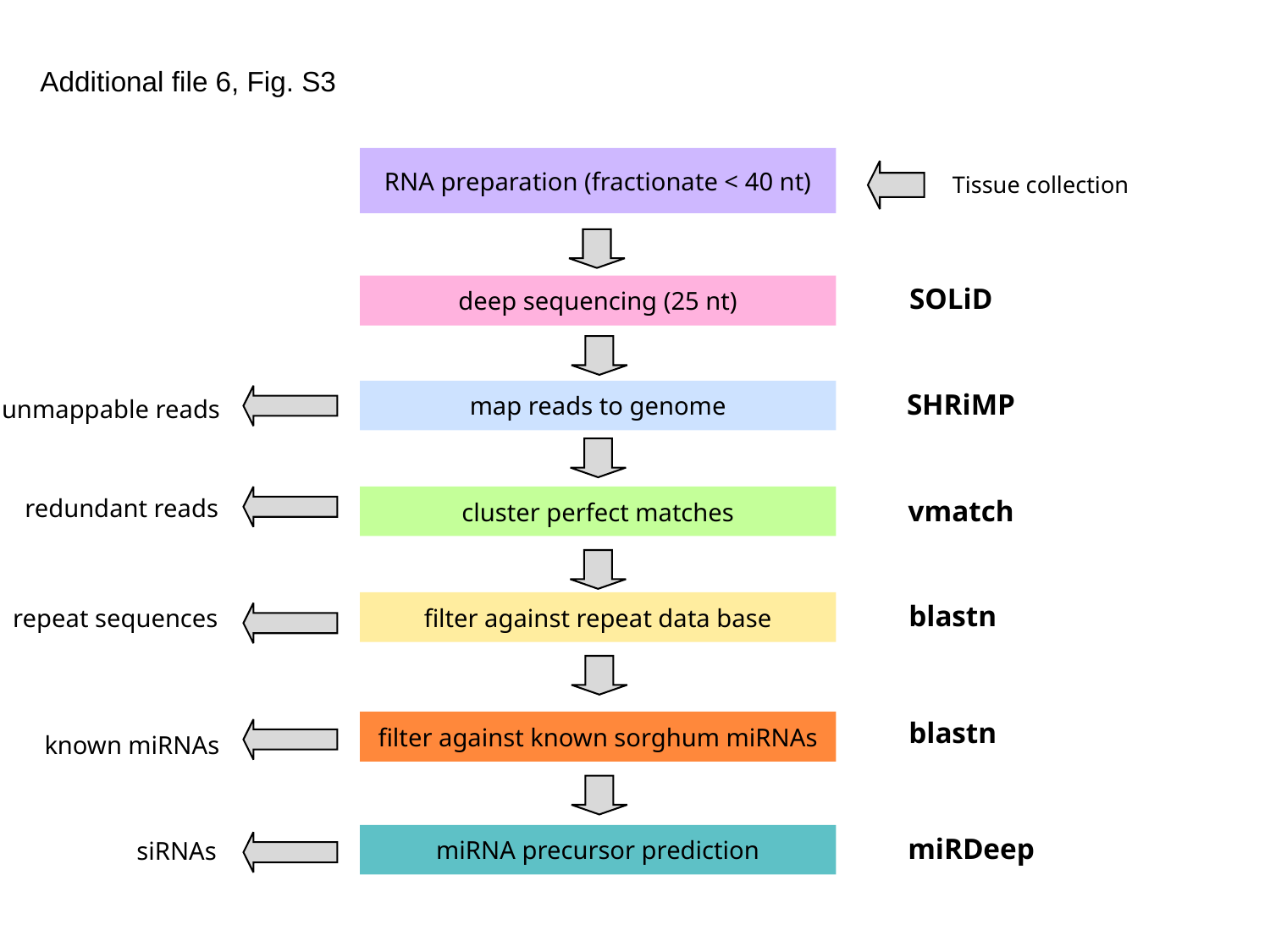

Additional file 6, Fig. S3
RNA preparation (fractionate < 40 nt)
Tissue collection
SOLiD
deep sequencing (25 nt)
map reads to genome
SHRiMP
unmappable reads
redundant reads
cluster perfect matches
vmatch
filter against repeat data base
blastn
repeat sequences
blastn
filter against known sorghum miRNAs
known miRNAs
miRDeep
miRNA precursor prediction
siRNAs
