# Supplementary material for: Characterization of the small RNA component of the transcriptome from grain and sweet sorghum stems
Source: BMC Genomics. 2011 Jul 8;12:356. doi: 10.1186/1471-2164-12-356 (PMC3143107; doi:10.1186/1471-2164-12-356)

sbi-MIR5381

```
gctttaaaccaaagcTCGGCGCTAAGATCTCTGGctccgacctcggcgccAAGATCTGTGGCGCCGAGCtcgacgccaggccacatggcgccttggtcagcgcccagatc
     AAACCAAAGCTCGGCGCTAAGA,bc02,1
       ACCAAAGCTCGGCGCTAAGA,bc01,1
                     CTAAGATCTCTGGCTCCGACCTCG,bc04,1
                        AGATCTCTGGCTCCGACC,bc05,1
                                   TCCGACCTCGGCGCCAAGATCTG,bc04,1
                                   TCCGACCTCGGCGCCAAGATCTG,bc05,1
                                      GACCTCGGCGCCAAGATC,bc01,1
                                      GACCTCGGCGCCAAGATCT,bc05,1
                                       AGCTCGGCGCCAAGATCTGTGGC,bc01,1
                                        GCTCGGCGCCAAGATCTGT,bc04,1
                                         CTCGGCGCCAAGATCTGTG,bc05,1
                                          TCGGCGCCAAGATCTGTGGC,bc05,1
                                                 CAAGATCTGTGGCGCCGA,bc01,1
                                                 CAAGATCTGTGGCGCCGAG,bc05,1
                                                   AGATCTGTGGCGCCGAGCTC,bc01,1
                                                   AGATCTGTGGCGCCGAGCTCG,bc01,1
                                                   AGATCTGTGGCGCCGAGCT,bc03,1
                                                   AGATCTGTGGCGCCGAGCTC,bc03,1
                                                   AGATCTGTGGCGCCGAGCTC,bc04,1
                                                   AGATCTGTGGCGCCGAGCTCG,bc04,1
                                                   AGATCTGTGGCGCCGAGCTC,bc05,3
                                                    GATCTGTGGCGCCGAGCTC,bc01,1
                                                    GATCTGTGGCGCCGAGCTC,bc04,1
                                                    GATCTGTGGCGCCGAGCTC,bc05,1
                                                    GATCTGTGGCGCCGAGCTCG,bc05,1
                                                     ATCTGTGGCGCCGAGCTC,bc01,1
                                                     ATCTGTGGCGCCGAGCTC,bc04,1
                                                                 GAGCTCGACGCCAGGCCACATG,bc01,1
                                                                 GAGCTCGACGCCAGGCCACATGG,bc02,1
                                                                 GAGCTCGACGCCAGGCCACA,bc04,1
                                                                 GAGCTCGACGCCAGGCCACATG,bc04,2
                                                                 GAGCTCGACGCCAGGCCACATGG,bc04,1
                                                                 GAGCTCGACGCCAGGCCACATG,bc05,1
                                                                 GAGCTCGACGCCAGGCCACATGG,bc05,1
                                                                          GCCAGGCCACATGGCGCCTTG,bc01,1
                                                                             AGGCCACATGGCGCCTTG,bc04,2
                                                                             AGGCCACATGGCGCCTTG,bc05,1
                                                                               GCCACATGGCGCCTTGGTCAGCGCC,bc04,1
```

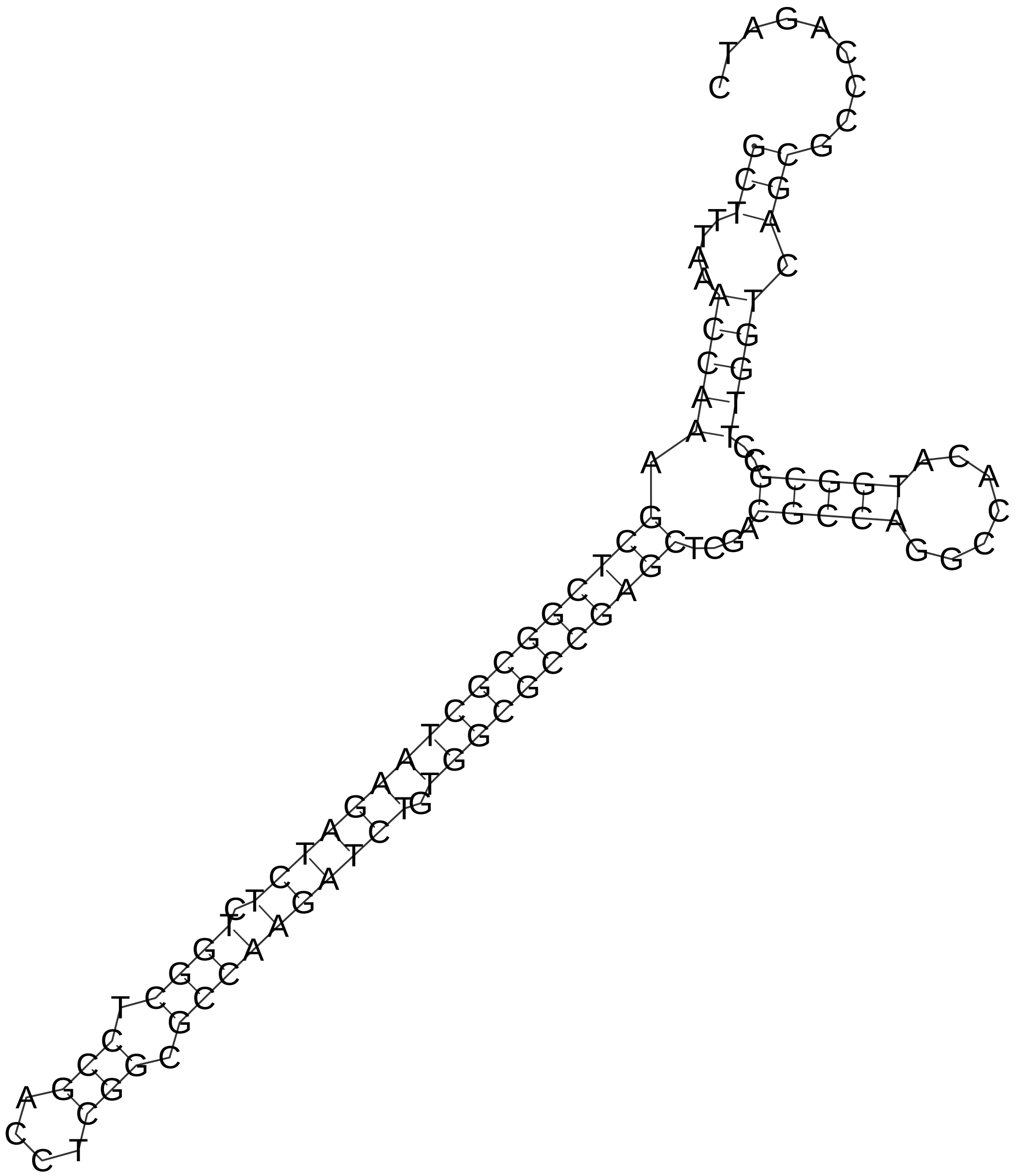

sbi-MIR5382

```
ttttcattgCCAATCTAAACAGGCCCTaactcatcagtggacattttgcaagaggagaaagccgaaagcctcttttgagctggGACCTGTTTAGATTGGGAacgaaaacg
  TTCATTGCCAATCTAAACAGGC,bc05,1
  TTCATTGCCAATCTAAACAGGCCC,bc05,1
   TCATTGCCAATCTAAACAGGCCC,bc05,1
    CATTGCCAATCTAAACAGGCCCT,bc02,1
    CATTGCCAATCTAAACAGGCCC,bc04,1
    CATTGCCAATCTAAACAGGCCCT,bc04,1
    CATTGCCAATCTAAACAGGCCCT,bc05,1
     ATTGCCAATCTAAACAGGCCCT,bc01,1
     ATTGCCAATCTAAACAGGCCCT,bc02,1
     ATTGCCAATCTAAACAGGCCC,bc03,1
     ATTGCCAATCTAAACAGGCCC,bc04,1
     ATTGCCAATCTAAACAGGCCCT,bc04,2
     ATTGCCAATCTAAACAGGCCCT,bc05,1
      TTGCCAATCTAAACAGGCCCT,bc01,1
      TTGCCAATCTAAACAGGCCCT,bc02,2
      TTGCCAATCTAAACAGGCCC,bc05,1
      TTGCCAATCTAAACAGGCCCT,bc05,1
        GCCAATCTAAACAGGCCCT,bc04,1
        GCCAATCTAAACAGGCCCT,bc04,2
        GCCAATCTAAACAGGCCC,bc05,1
        GCCAATCTAAACAGGCCCT,bc05,1
         CCAATCTAAACAGGCCCTAAC,bc03,2
           AATCTAAACAGGCCCTAACT,bc01,1
           AATCTAAACAGGCCCTAACT,bc03,1
                    AGGCCCTAACTCATCAGTGGACAT,bc05,1
                       CCCTAACTCATCAGTGGACATTT,bc05,1
                             CTCATCAGTGGACATTTTGCAAGA,bc05,1
                                ATCAGTGGACATTTTGCAAGAGGA,bc01,1
                                  CAGTGGACATTTTGCAAGAGGAG,bc05,1
                                      GGACATTTTGCAAGAGGAGAAAG,bc05,1
                                         CATTTTGCAAGAGGAGAAAGCC,bc05,1
                                                                 AAGCCTCTTTTGAGCTGGGACCT,bc01,2
                                                                  AGCCTCTTTTGAGCTGGGACC,bc05,1
                                                                                  GGACCTGTTTAGATTGGGAAC,bc03,1
                                                                                  GGACCTGTTTAGATTGGGA,bc04,1
                                                                                  GGACCTGTTTAGATTGGG,bc05,1
                                                                                  GGACCTGTTTAGATTGGGA,bc05,1
                                                                                  GGACCTGTTTAGATTGGGAA,bc05,1
                                                                                  GGACCTGTTTAGATTGGGAAC,bc05,1
                                                                                  GGACCTGTTTAGATTGGGAACG,bc05,2
                                                                                  GGACCTGTTTAGATTGGGAACGAA,bc05,1
                                                                                   GACCTGTTTAGATTGGGAACGAAA,bc03,1
                                                                                     CCTGTTTAGATTGGGAACGA,bc01,1
                                                                                     CCTGTTTAGATTGGGAACGAAAA,bc01,1
                                                                                     CCTGTTTAGATTGGGAACGA,bc02,1
                                                                                     CCTGTTTAGATTGGGAACGA,bc03,1
                                                                                     CCTGTTTAGATTGGGAACGAA,bc03,1
                                                                                     CCTGTTTAGATTGGGAACGAAAA,bc03,1
                                                                                     CCTGTTTAGATTGGGAAC,bc04,8
                                                                                     CCTGTTTAGATTGGGAACG,bc04,3
                                                                                     CCTGTTTAGATTGGGAACGAAAA,bc04,2
                                                                                     CCTGTTTAGATTGGGAAC,bc05,3
                                                                                     CCTGTTTAGATTGGGAACG,bc05,2
                                                                                     CCTGTTTAGATTGGGAACGA,bc05,1
                                                                                     CCTGTTTAGATTGGGAACGAA,bc05,2
                                                                                     CCTGTTTAGATTGGGAACGAA,bc05,2
                                                                                     CCTGTTTAGATTGAGAACGAAAA,bc05,2
                                                                                     CCTGTTTAGATTGGGAACGAAAA,bc05,1
                                                                                     CCTGTTTAGATTGGGAACGAAAA,bc05,1
```

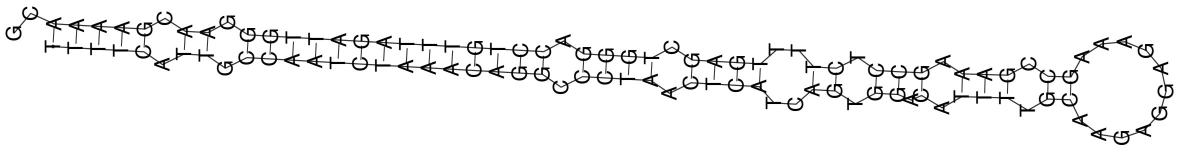

sbi-MIR5383

```
tggacctatttataggccggccATGACAGAGCTCCGGCAGAGATATttTTCTCCGCCGAGCTTATCTGTGGcgctagttcactgccatctggccgaggtggtctggcata
                 CGGCCATGACAGAGCTCCG,bc02,1
                  GGCCATGACAGAGCTCCGGCAGA,bc04,1
                      ATGACAGAGCTCCGGCAGAGA,bc01,1
                      ATGACAGAGCTCCGGCAGAGATAT,bc01,2
                      ATGACAGAGCTCCGGCAGAGATAT,bc02,2
                      ATGACAGAGCTCCGGCAGAGATAT,bc04,2
                      ATGACAGAGCTCCGGCAGAGATAT,bc05,5
                           AGAGCTCCGGCAGAGATA,bc04,1
                                                 TCTCCGCCGAGCTTATCTGTGGCG,bc04,1
                                                   TCCGCCGAGCTTATCTGTGGCGCT,bc02,1
                                                                                    CCATCTGGCCGAGGTGGTCTGGC,bc02,1
```

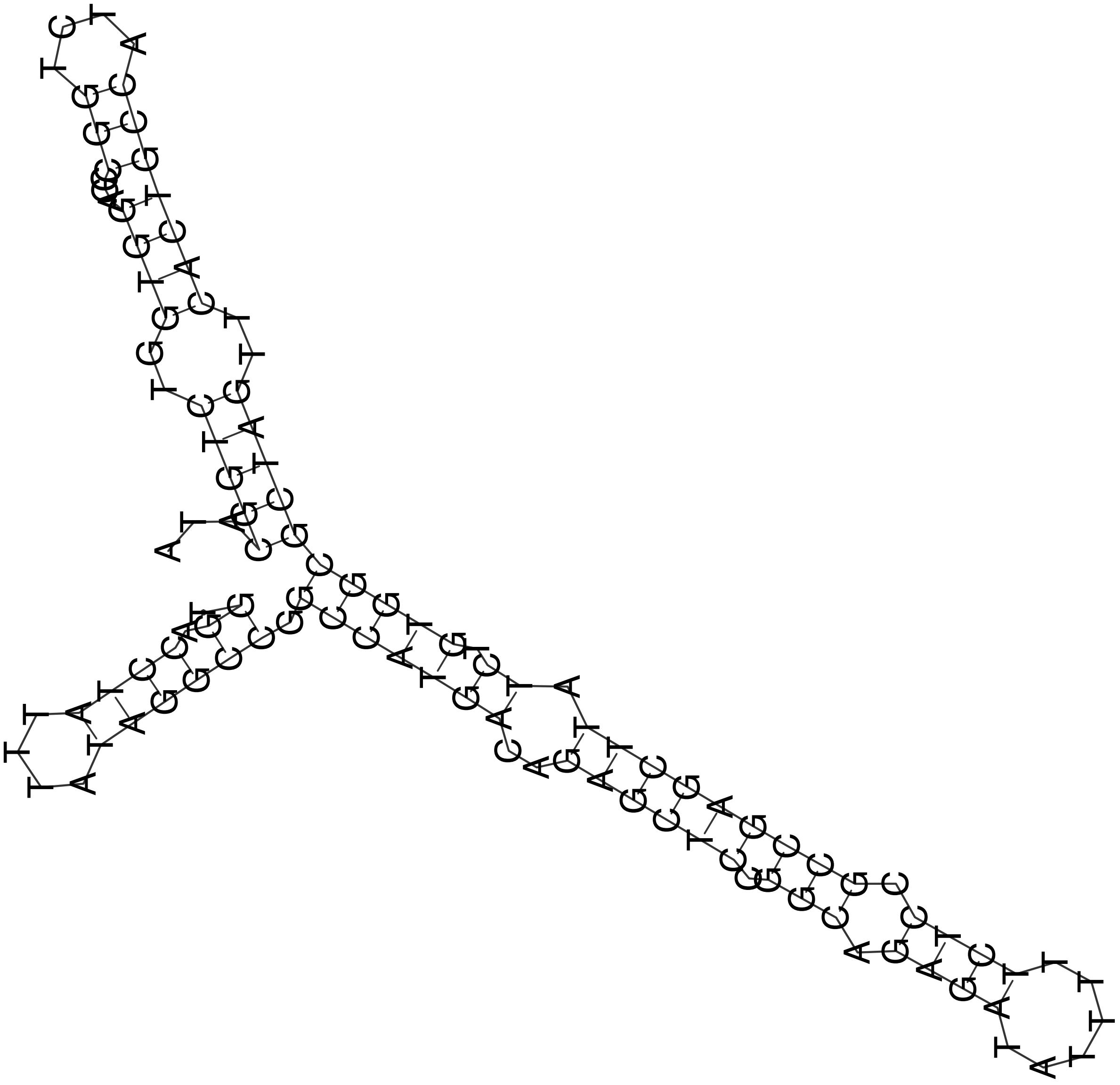

sbi-MIR5384

```
ggggaaccaggaagcggcgccgccgccgggccCTTGGCCGGTGCACGCGTCcacccgagggttgggCGCGCCGCCGTCCAGCGGctccgtgacggcgccaaaaccggtgc
                         CCGGGCCCTTGGCCGGTG,bc05,1
                          CGGGCCCTTGGCCGGTGC,bc03,1
                          CGGGCCCTTGGCCGGTGCGCGCG,bc05,1
                                 TTGGCCGGTGCGCGCGTCCACCC,bc04,3
                                 TTGGCCGGTGCGCGCGTCCACCCG,bc04,1
                                 TTGGCCGGTGCGCGCGTCCACCCGA,bc04,1
                                 TTGGCCGGTGCGCGCGTCCACCC,bc05,1
                                  TGGCCGGTGCGCGCGTCCACCCG,bc05,1
                                    GCCGGTGCGCGCGTCCACCCGAGGG,bc03,1
                                      CGGTGCGCGCGTCCACCCGAGGGT,bc03,1
                                       GGTGCGCGCGTCCACCCGAGGGTT,bc04,1
                                                         AGGGTTGGGCGCGCCGCC,bc05,1
                                                                 GCGCGCCGCCGTCCAGCGG,bc05,1
                                                                 GCGCGCCGCCGTCCAGCGGC,bc05,1
                                                                  CGCGCCGCCGTCCAGCGGCT,bc01,1
                                                                  CGCGCCGCCGTCCAGCGGCTCC,bc01,1
                                                                  CGCGCCGCCGTCCAGCGG,bc02,2
                                                                  CGCGCCGCCGTCCAGCGG,bc04,3
                                                                  CGCGCCGCCGTCCAGCGG,bc05,3
                                                                  CGCGCCGCCGTCCAGCGGC,bc05,1
                                                                  CGCGCCGCCGTCCAGCGGCTCCGT,bc05,1
                                                                    CGCCGCCGTCCAGCGGCT,bc02,1
```

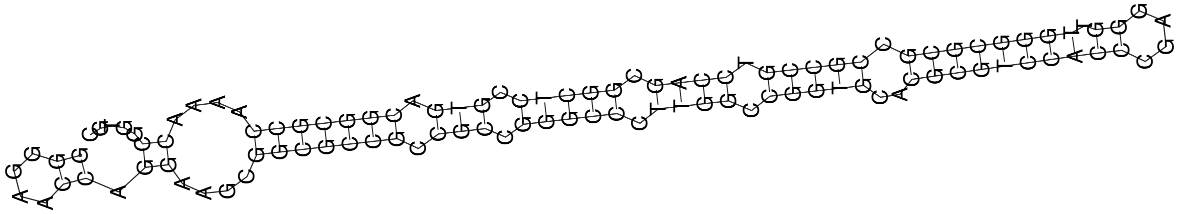

sbi-MIR5385

```
ggagttttgcacgccgccgcttctcACCACCAACCCCACCGCTTCTCcgcgccgaggggcgaGAAGCGGTGGTGTTGGTGGTGAgaagcagcggcgtggcagatttgcag
                     TCTCACCACCAACCCCACCG,bc03,3
                     TCTCACCACCAACCCCACCGC,bc03,1
                     TCTCACCACCAACCCCACC,bc04,14
                     TCTCACCACCAACCCCACCG,bc04,8
                     TCTCACCACCAACCCCACCGC,bc04,8
                     TCTCACCACCAACCCCAC,bc05,4
                     TCTCACCACCAACCCCACC,bc05,28
                     TCTCACCACCAACCCCACCG,bc05,5
                     TCTCACCACCAACCCCACCGC,bc05,7
                      CTCACCACCAACCCCACCGC,bc01,1
                      CTCACCACCAACCCCACCGCT,bc01,1
                      CTCACCACCAACCCCACCG,bc03,3
                      CTCACCACCAACCCCACC,bc04,1
                      CTCACCACCAACCCCACC,bc04,2
                      CTCACCACCAACCCCACCG,bc04,2
                      CTCACCACCAACCCCACCGC,bc04,2
                      CTCACCACCAACCCCACCGCT,bc04,3
                      CTCACCACCAACCCCACC,bc05,3
                      CTCACCACCAACCCCACCGCT,bc05,2
                       TCACCACCAACCCCACCGCTT,bc01,1
                       TCACCACCAACCCCACCGC,bc04,1
                       TCACCACCAACCCCACCGC,bc05,2
                       TCACCACCAACCCCACCGCT,bc05,1
                        CACCACCAACCCCACCGCTT,bc01,1
                        CACCACCAACCCCACCGCTTC,bc03,1
                        CACCACCAACCCCACCGC,bc04,5
                        CACCACCAACCCCACCGCT,bc04,1
                        CACCACCAACCCCACCGCTTC,bc05,9
                         ACCACCAACCCCACCGCT,bc02,1
                         ACCACCAACCCCACCGCTTC,bc04,11
                         ACCACCAACCCCACCGCTT,bc05,1
                          CCACCAACCCCACCGCTTC,bc03,1
                          CCACCAACCCCACCGCTTC,bc05,6
                           CACCAACCCCACCGCTTC,bc01,2
                           CACCAACCCCACCGCTTC,bc03,1
                                                        GGGCGAGAAGCGGTGGTGTTGGTGG,bc03,1
                                                        GGGCGAGAAGCGGTGGTGTTGGTGG,bc04,3
                                                        GGGCGAGAAGCGGTGGTGTTGGTGG,bc05,3
                                                         GGCGAGAAGCGGTGGTGTTGGTGGT,bc01,1
                                                         GGCGAGAAGCGGTGGTGTTGGTGG,bc05,2
                                                          GCGAGAAGCGGTGGTGTTGGTGG,bc01,1
                                                            GAGAAGCGGTGGTGTTGGTGG,bc01,3
                                                            GAGAAGCGGTGGTGTTGGTGG,bc03,3
                                                            GAGAAGCGGTGGTGTTGGTGG,bc04,8
                                                            GAGAAGCGGTGGTGTTGGTGG,bc05,16
                                                             AGAAGCGGTGGTGTTGGTGG,bc01,4
                                                             AGAAGCGGTGGTGTTGGTGG,bc03,1
                                                             AGAAGCGGTGGTGTTGGTGG,bc05,4
                                                              GAAGCGGTGGTGTTGGTGG,bc01,8
                                                              GAAGCGGTGGTGTTGGTGG,bc03,9
                                                              GAAGCGGTGGTGTTGGTGGTGA,bc03,1
                                                              GAAGCGGTGGTGTTGGTGG,bc04,11
                                                              GAAGCGGTGGTGTTGGTGGTG,bc04,1
                                                              GAAGCGGTGGTGTTGGTGG,bc05,23
                                                              GAAGCGGTGGTGTTGGTGGTG,bc05,1
                                                               AAGCGGTGGTGTTGGTGG,bc01,2
                                                               AAGCGGTGGTGTTGGTGG,bc02,1
                                                               AAGCGGTGGTGTTGGTGG,bc03,2
                                                               AAGCGGTGGTGTTGGTGG,bc04,10
                                                               AAGCGGTGGTGTTGGTGGTG,bc04,1
                                                               AAGCGGTGGTGTTGGTGG,bc05,8
                                                               AAGCGGTGGTGTTGGTGGTG,bc05,1
                                                               AAGCGGTGGTGTTGGTGGTGAG,bc05,1
                                                                AGCGGTGGTGTTGGTGGTGAG,bc01,1
                                                                AGCGGTGGTGTTGGTGGTG,bc02,1
                                                                AGCGGTGGTGTTGGTGGTG,bc04,9
                                                                AGCGGTGGTGTTGGTGGTGA,bc04,1
                                                                AGCGGTGGTGTTGGTGGTGAG,bc04,1
                                                                AGCGGTGGTGTTGGTGGTGAGA,bc04,1
                                                                AGCGGTGGTGTTGGTGGTG,bc05,7
                                                                 GCGGTGGTGTTGGTGGTG,bc01,4
                                                                 GCGGTGGTGTTGGTGGTGAGA,bc01,1
                                                                 GCGGTGGTGTTGGTGGTG,bc03,1
                                                                 GCGGTGGTGTTGGTGGTGAGA,bc03,2
                                                                 GCGGTGGTGTTGGTGGTG,bc04,16
                                                                 GCGGTGGTGTTGGTGGTGA,bc04,1
                                                                 GCGGTGGTGTTGGTGGTGAGA,bc04,4
                                                                 GCGGTGGTGTTGGTGGTG,bc05,17
                                                                 GCGGTGGTGTTGGTGGTGA,bc05,1
                                                                 GCGGTGGTGTTGGTGGTGAG,bc05,1
                                                                 GCGGTGGTGTTGGTGGTGAGA,bc05,3
                                                                  CGGTGGTGTTGGTGGTGAGA,bc03,2
                                                                  CGGTGGTGTTGGTGGTGAGA,bc04,4
                                                                  CGGTGGTGTTGGTGGTGAGA,bc05,5
                                                                   GGTGGTGTTGGTGGTGAGA,bc01,5
                                                                   GGTGGTGTTGGTGGTGAGA,bc03,1
                                                                   GGTGGTGTTGGTGGTGAG,bc04,1
                                                                   GGTGGTGTTGGTGGTGAG,bc04,1
                                                                   GGTGGTGTTGGTGGTGAGA,bc04,1
                                                                   GGTGGTGTTGGTGGTGAGA,bc05,8
                                                                    GTGGTGTTGGTGGTGAGA,bc01,4
                                                                    GTGGTGTTGGTGGTGAGA,bc03,7
                                                                    GTGGTGTTGGTGGTGAGAA,bc03,1
                                                                    GTGGTGTTGGTGGTGAGA,bc04,5
                                                                    GTGGTGTTGGTGGTGAGA,bc05,10
                                                                    GTGGTGTTGGTGGTGAGA,bc05,1
                                                                      GGTGTTGGTGGTGAGAAGCA,bc01,1
                                                                      GGTGTTGGTGGTGAGAAGCAG,bc05,1
                                                                         GTTGGTGGTGAGAAGCAGCGG,bc03,2
                                                                          TTGGTGGTGAGAAGCAGCGGCG,bc05,1
```

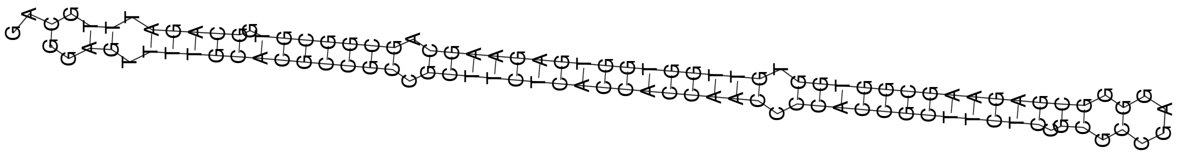

sbi-MIR5386

```
cgcactgtgggtatgCGTCGCTGTCGCGCGCTGcaggccatgttccatggcttcctgtcaGGTCAGGGCAGAGCACGCAcccctcgtcttgctttgggcatgtctcca
                                                             GTCAGGGCAGAGCACGCACCCC,bc01,1
                                                             GTCAGGGCAGAGCACGCACCCCT,bc01,1
                                                             GTCAGGGCAGAGCACGCACCCCTCG,bc01,5
                                                             GTCAGGGCAGAGCACGCACCCCTCG,bc02,5
                                                             GTCAGGGCAGAGCACGCACCCCTCG,bc03,4
                                                             GTCAGGGCAGAGCACGCAC,bc04,1
                                                             GTCAGGGCAGAGCACGCACCC,bc04,1
                                                             GTCAGGGCAGAGCACGCACCCC,bc04,4
                                                             GTCAGGGCAGAGCACGCACCCCT,bc04,9
                                                             GTCAGGGCAGAGCACGCACCCCTC,bc04,1
                                                             GTCAGGGCAGAGCACGCACCCCTCG,bc04,30
                                                             GTCAGGGCAGAGCACGCACCCC,bc05,1
                                                             GTCAGGGCAGAGCACGCACCCCT,bc05,2
                                                             GTCAGGGCAGAGCACGCACCCCTC,bc05,1
                                                             GTCAGGGCAGAGCACGCACCCCTCG,bc05,4
                                                              TCAGGGCAGAGCACGCACCCCT,bc04,1
```

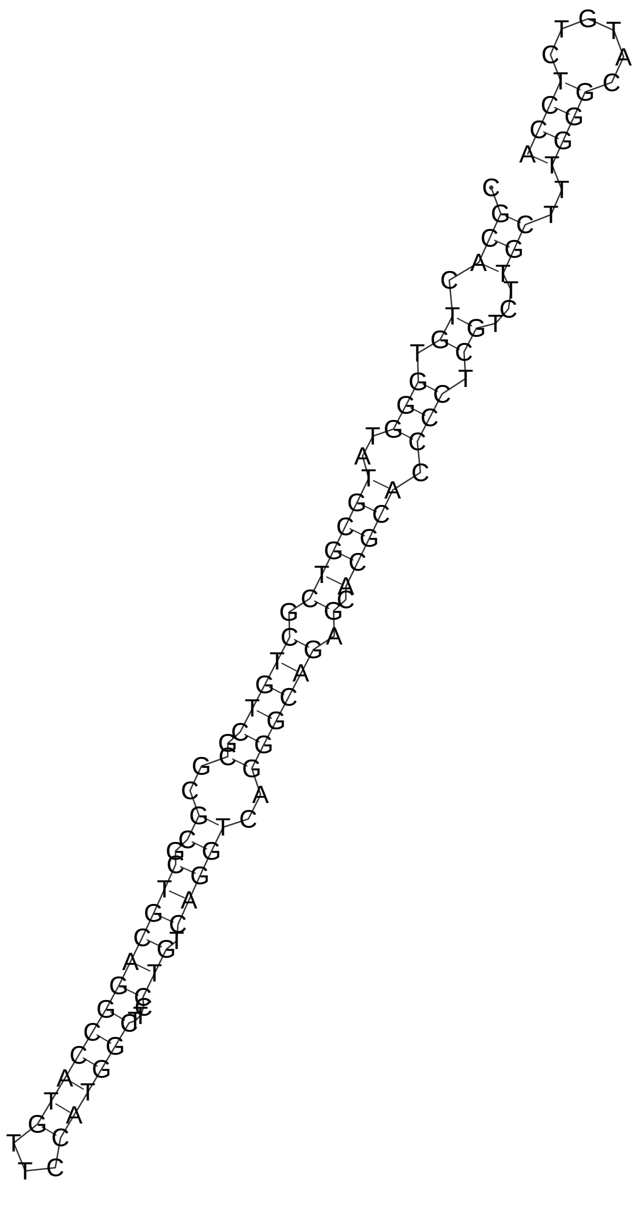

sbi-MIR5387

```
tcgTAACACGAACCGGTGCTAAAGGATCttgcccaacggctactgacagctgttgttggggcagggaCCCTTTAGCACCGGTTCGTGTTACAaaccggtggtaaagggtt
 CGTAACACGAACCGGTGCTAAA,bc03,1
  GTAACACGAACCGGTGCT,bc02,1
  GTAACACAAACCGGTGCTAAAG,bc05,1
    AACACGAACCGGTGCTAA,bc03,1
     ACACGAACCGGTGCTAAAGGA,bc03,1
     ACACGAACCGGTGCTAAAGGA,bc05,1
      CACGAACCGGTGCTAAAG,bc05,1
      CACGAACCGGTGCTAAAGGATC,bc05,1
       ACGAACCGGTGCTAAAGGA,bc01,1
       ACGAACCGGTGCTAAAGGAT,bc04,1
        CGAACCGGTGCTAAAGGA,bc01,1
         GAACCGGTGCTAAAGGATC,bc05,1
          AACCGGTGCTAAAGGATC,bc04,1
                  CTAAAGGGTCTTGCCCAACGGCT,bc02,1
                  CTAAAGGATCCTGCCCAACGGCT,bc04,2
                     AAGGATCTTGCCCAACGGCTACTG,bc04,1
                      AGGATCTTGCCCAACGGCTACTGAC,bc02,1
                       GGATCTTGCCCAACGGCTACTGA,bc02,1
                       GGATCTTGCCCAACGGCTACTGAC,bc02,1
                         ATCTTGCCCAACGGCTACTGACAGC,bc05,1
                                         ACTGACAGCTGTTGTTGGGGCAGGG,bc03,1
                                            GACAGCTGTTGTTGGGGCAG,bc05,1
                                                GCTGTTGTTGGGGCAGGGACCC,bc05,1
                                                      GTTGGGGCAGGGACCCTTTAGCA,bc03,1
                                                      GTTGGGGCAGGGACCCTT,bc04,1
                                                        TGGGGCAGGGACCCTTTAG,bc03,1
                                                          GGGCAGGGATCCTTTAGCACCGG,bc04,1
                                                           GGCAGGGACCCTTTAGCACCGGTT,bc04,1
                                                            GCAGGGACCCTTTAGCACCGGTTCG,bc02,1
                                                             CAGGGACCCTTTAGCACCGGTTCGT,bc02,1
                                                             CAGGGACCCTTTAGCACCGG,bc04,1
                                                             CAGGGACCCTTTAGCACCG,bc05,1
                                                             CAGGGACCCTTTAGCACCGGTTC,bc05,2
                                                              AGGGACCCTTTAGCACCGGTTCGT,bc04,1
                                                              AGGGACCCTTTAGCACCGGTTCG,bc05,1
                                                               GGGACCCTTTAGCACCGG,bc03,1
                                                               GGGACCCTTTAGCACCGGTTCGTGT,bc03,1
                                                               GGGACCCTTTAGCACCGGTTCG,bc05,1
                                                               GGGACCCTTTAGCACCGGTTCGTGT,bc05,5
                                                                GGACCCTTTAGCACCGGTTCGTGT,bc04,1
                                                                 GACCCTTTAGCACCGGTTCGTGT,bc03,1
                                                                 GACCCTTTAGCACCGGTTCGTGTT,bc04,1
                                                                 GACCCTTTAGCATCGGTTCGTGT,bc05,1
                                                                 GACCCTTTAGCACCGGTTCATGTT,bc05,1
                                                                 GACCCTTTAGCACCGGTTCGTGTTA,bc05,1
                                                                  ACCCTTTAGCACCGGTTCGTGTTAC,bc05,1
                                                                   CCCTTTAGCACCGGTTCGTGTTAC,bc04,1
                                                                    CCTTTAGCACCGGTTCGTGT,bc04,1
                                                                    CCTTTAGCACCGGTTCGTGTTAC,bc04,1
                                                                    CCTTTAGCACCGGTTCGTGTT,bc05,1
                                                                     CTTTAGCACCGGTTCGTGT,bc04,1
                                                                     CTTTAGCACCGGTTCGTGT,bc05,1
                                                                        TAGCACCGGTTCGTGTTAC,bc01,1
                                                                         AGCACCGGTTCGTGTTACAA,bc04,1
                                                                         AGCACCGGTTCGTGTTACCAACCG,bc04,1
                                                                         AGCACCGGTTCGTGTTACCAACCG,bc05,3
                                                                          GCACCGGTTCGTGTTACAAACC,bc03,1
                                                                             CCGGTTCGTGTTACAAAC,bc01,1
                                                                             CCGGTTCGTGTTACAAAC,bc05,1
                                                                              CGGTTCGTGTTACAAACCGGTG,bc02,1
                                                                               GGTTCGTGTTACAAACCGGTG,bc03,2
                                                                               GGTTCGTGTTACAAACCGGTG,bc04,1
                                                                               GGTTCGTGTTACAAACCGG,bc05,1
                                                                               GGTTCGTGTTACAAACCGGTG,bc05,1
                                                                              CGGCCCGTGTTACAAACCGGTG,bc02,1
                                                                                   CGTGTTACAAACCGGTGCTAAAGGG,bc04,1
                                                                                    GTGTTACAAACCGGTGCTAAAGGGT,bc05,1
```

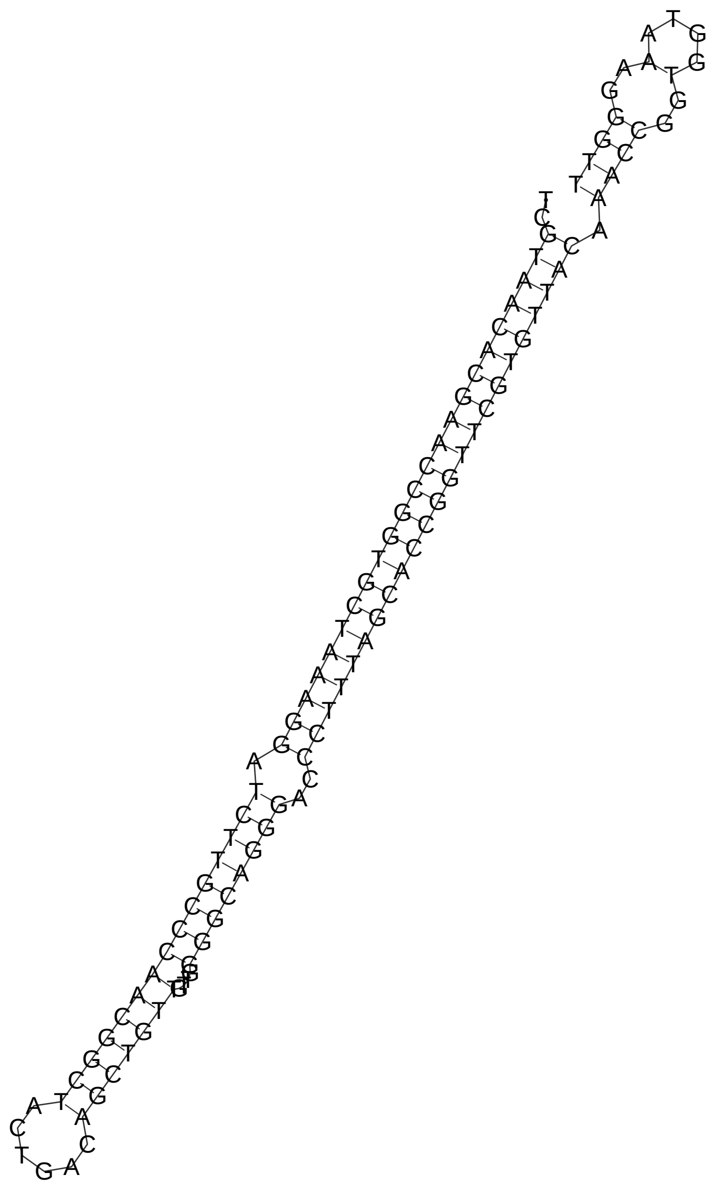

sbi-MIR5388

```
aaaaaaaaatctttgccgggagggtcctCAGCAAACATTCGGCAAAGAAAAtttgaaaaaaaattagaaaATCTTTGCCGGGTGTCTCTGACacagcttccggcaaagca
               CCGGGAGGGTCCTCAGCAAACATT,bc04,1
                 GGGAGGGTCCTCAGCAAACATTCG,bc05,1
                  GGAGGGTCCTCAGCAAACA,bc04,3
                     GGGTCCTCAGCAAACATTCGGC,bc04,1
                     GGGTCCTCAGCAAACATTCGGCAAA,bc05,1
                      GGTCCTCAGCAAACATTCGGCAA,bc04,1
                       GTCCTCAGCAAACATTCGGC,bc04,1
                        TCCTCAGCAAACATTCGGC,bc04,1
                         CCTCAGCAAACATTCGGC,bc04,1
                         CCTCAGCAAACATTCGGCAAAGA,bc05,1
                          CTCAGCAAACATTCGGCAAAGAAAA,bc05,1

                                                                 AGAAAATCTTTGCCGGGTG,bc04,1
                                                                 AGAAAATCTTTGCCGGGTG,bc05,1
                                                                  GAAAATCTTTGCCGGGTG,bc04,1
                                                                   AAAATCTTTGCCGGGTGTC,bc04,1
                                                                    AAATCTTTGCTGGGTGTCTCTG,bc05,1
                                                                      ATCTTTGCCGGGTGTCTCTGAC,bc05,1
                                                                          TTGCCGGGTGTCTCTGACACAGCT,bc02,1
                                                                          TTGCTGGGTGTCTCTGACACAGCT,bc04,1
                                                                            GCCGGGTGTCTCTGACACAGCTTCC,bc02,1
                                                                            GCCGGGTGTCTCTGACACAGCTTCC,bc04,1
                                                                             CCGGGTGTCTCTGACACAGCTTC,bc05,1
                                                                               GGGTGTCTCTGACACAGCT,bc03,1
                                                                               GGGTGTCTCTGACACAGCTTC,bc05,1
                                                                                   GTCTCTGACACAGCTCCCGGCAAA,bc04,1
```

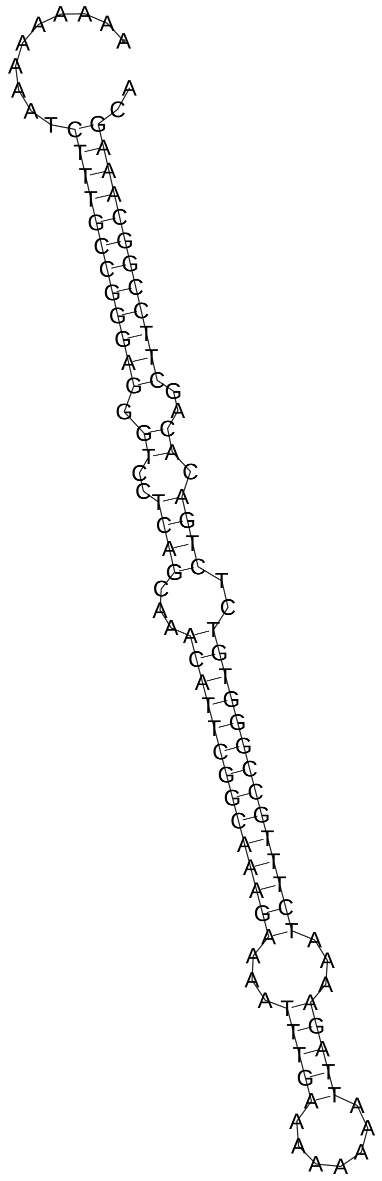

sbi-MIR5389

```
tgcagccacaaacatatgctgcaagtactaatgctttgttcGCTTGAGTTTATCAGCCGAGTctgaacactactttaatttcagccATGGCTTATCAGCCAAGTGAacag
                TGCTGCAAGTACTAATGCTTTGTT,bc02,1
                          ACTAATGCTTTGTTCGCT,bc05,1
                              ATGCTTTGTTCGCTTGAG,bc04,1
                                  TTTGTTCGCTTGAGTTTA,bc01,1
                                  TTTGTTCGCTTGAGTTTAT,bc04,1
                                  TTTGTTCGCTTGAGTTTATC,bc04,2
                                  TTTGTTCGCTTGAGTTTA,bc05,1
                                  TTTGTTCGCTTGAGTTTATC,bc05,1
                                   TTGTTCGCTTGAGTTTATC,bc01,1
                                   TTGTTCGCTTGAGTTTATC,bc05,2
                                    TGTTCGCTTGAGTTTATCAGCCG,bc02,3
                                    TGTTCGCTTGAGTTTATCAGC,bc04,1
                                    TGTTCGCTTGAGTTTATCTGCC,bc04,1
                                    TGTTCGCTTGAGTTTATCAGCCG,bc05,2
                                    TGTTCGCTTGAGTTTATCAGCCGA,bc05,2
                                     GTTCGCTTGAGTTTATCAGCCGAGT,bc01,1
                                     GTTCGCTTGAGTTTATCAGCC,bc02,1
                                     GTTCGCTTGAGTTTATCAGCCGA,bc03,1
                                     GTTCGCTTGAGTTTATCAG,bc04,1
                                     GTTCGCTTGAGTTTATCA,bc05,1
                                     GTTCGCTTGAGTTTATCAGCC,bc05,1
                                     GTTCGCTTGAGTTTATCAGCCG,bc05,1
                                     GTTCGCTTGAGTTTATCAGCCGA,bc05,1
                                     GTTCGCTTGACTTTATCAGCCGAG,bc05,1
                                      TTCGCTTGAGTTTATCAGCCGA,bc01,1
                                      TTCGCTTGAGTTTATCAG,bc05,1
                                      TTCGCTTGAGTTTATCAGCCG,bc05,1
                                       TCGCTTGAGTTTATCAGCC,bc01,1
                                       TCGCTTGAGTTTATCAGCC,bc02,1
                                       TCGCTTGAGTTTATCAGCC,bc04,2
                                       TCGCTTGAGTTTATCAGCCG,bc04,3
                                        CGCTTGAGTTTATCAGCCG,bc04,1
                                        CGCTTGAGTTTATCAGCCGA,bc05,1
                                         GCTTGAGTTTATCAGCCG,bc05,1
                                          CTTGAGTTTATCAGCCGA,bc03,1
                                           TTGAGTTTATCAGCCGAGTCT,bc04,1
                                                                GAACACTACTTTAATTTCAGCC,bc01,1
                                                                GAACACTACTTTAATTTCAGCCA,bc04,1
                                                                GAACACTACTTTAATTTCAGCCAT,bc04,1
                                                                   CACTACTTTAATTTCAGCCATGG,bc04,1
                                                                                  AGCCATGGCTTATCAGCCAA,bc05,1
                                                                                   GCCATGGCTTATCAGCCAAGT,bc04,1
```

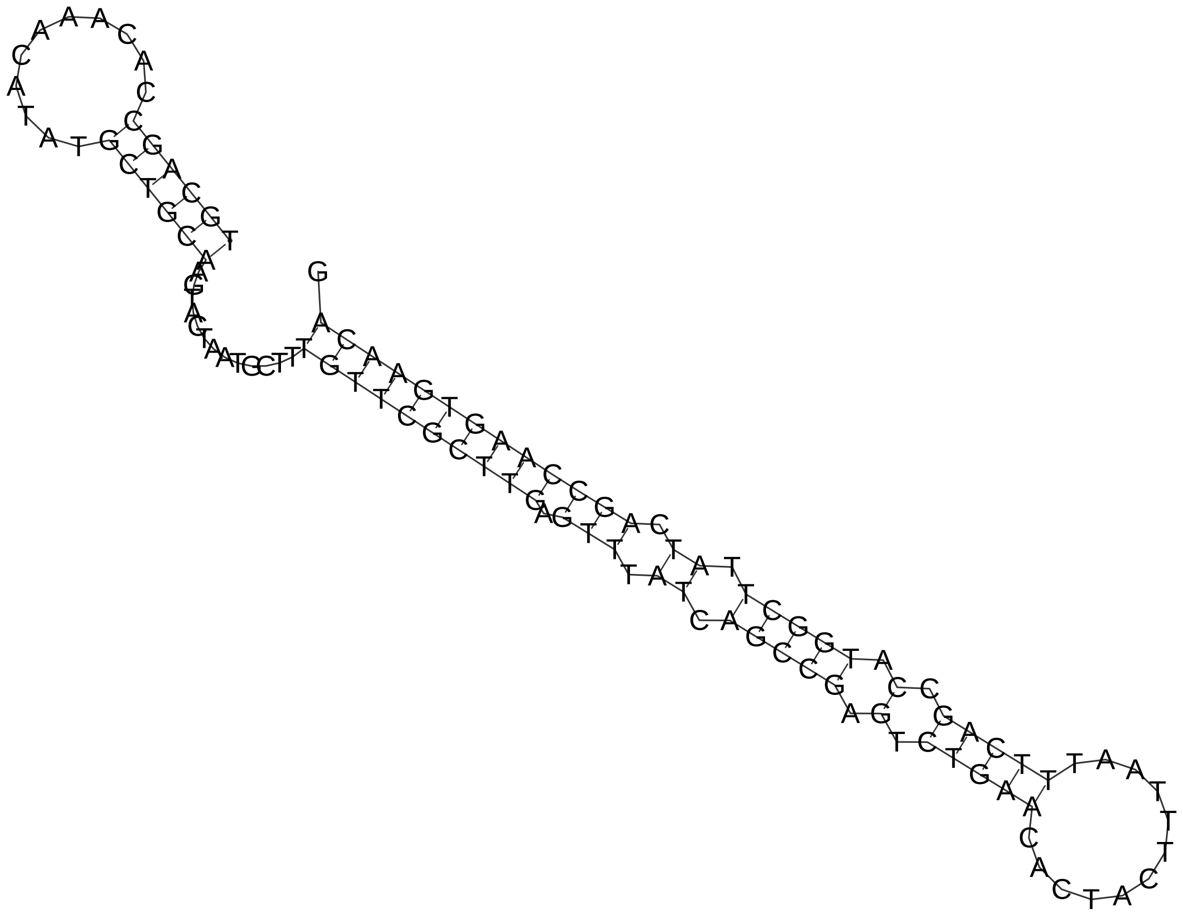

Supplement: Additional file 7 — Hairpin structures of the newly discovered miRNAs. Figure S4 presents a collection of hairpin structures from newly discovered miRNAs. Sequences are depicted together with the frequency distribution of the small RNA reads aligned to the hairpin. The 2D hairpin structure produced by the miRDeep software is also shown. [file 1471-2164-12-356-S7.PDF]
